# How Intrinsic Molecular Dynamics Control Intramolecular Communication in Signal Transducers and Activators of Transcription Factor STAT5

**DOI:** 10.1371/journal.pone.0145142

**Published:** 2015-12-30

**Authors:** Florent Langenfeld, Yann Guarracino, Michel Arock, Alain Trouvé, Luba Tchertanov

**Affiliations:** 1 Laboratoire de Biologie et Pharmacologie Appliquée Ecole Normale Supérieure de Cachan, CNRS, Université Paris-Saclay, Cachan, France; 2 Centre de Mathématiques et de Leurs applications, Ecole Normale Supérieure de Cachan, CNRS, Université Paris-Saclay, Cachan, France; University of Copenhagen, DENMARK

## Abstract

Signal Transducer and Activator of Transcription STAT5 is a key mediator of cell proliferation, differentiation and survival. While STAT5 activity is tightly regulated in normal cells, its constitutive activation directly contributes to oncogenesis and is associated with a broad range of hematological and solid tumor cancers. Therefore the development of compounds able to modulate pathogenic activation of this protein is a very challenging endeavor. A crucial step of drug design is the understanding of the protein conformational features and the definition of putative binding site(s) for such modulators. Currently, there is no structural data available for human STAT5 and our study is the first footprint towards the description of structure and dynamics of this protein. We investigated structural and dynamical features of the two STAT5 isoforms, STAT5a and STAT5b, taken into account their phosphorylation status. The study was based on the exploration of molecular dynamics simulations by different analytical methods. Despite the overall folding similarity of STAT5 proteins, the MD conformations display specific structural and dynamical features for each protein, indicating first, sequence-encoded structural properties and second, phosphorylation-induced effects which contribute to local and long-distance structural rearrangements interpreted as allosteric event. Further examination of the dynamical coupling between distant sites provides evidence for alternative profiles of the communication pathways inside and between the STAT5 domains. These results add a new insight to the understanding of the crucial role of intrinsic molecular dynamics in mediating intramolecular signaling in STAT5. Two pockets, localized in close proximity to the phosphotyrosine-binding site and adjacent to the channel for communication pathways across STAT5, may constitute valid targets to develop inhibitors able to modulate the function-related communication properties of this signaling protein.

## Introduction

The Signal Transducer and Activator of Transcription (STAT) proteins are a family of cytoplasmic transcriptional factors which transmit a broad spectrum of signals required to initiate many physiological processes. STAT proteins comprises seven isoforms–STAT1, STAT2, STAT3, STAT4, STAT5a, STAT5b and STAT6 –that mediate a cellular signal transfer from the cytoplasm to the DNA thus regulating the transcription of major genes relevant for normal or neoplastic cell growth or survival [1–3]. STAT transcription factors are activated by various kinases and act together with cell type-specific cofactors or co-repressors providing their cell-type specificity. As all STATs, STAT5 promotes the transcription of different specific genes, such as *Bcl-xL*, *cyclin D1/D2* or *Myc*, and thus is involved in, but not limited to, the physiological control of apoptosis, cell cycle progression and reactive oxygen species (ROS) production. In hematological malignancies, such as chronic myeloid leukemia (CML) and other myeloproliferative neoplasms (MPNs), as well as in solid tumors, it has been shown that overexpression of STAT5 and deregulation of its phosphorylation contribute to disease progression and mediate resistance to tyrosine kinase inhibitors (TKIs) resistance [4,5].

**Fig 1:**
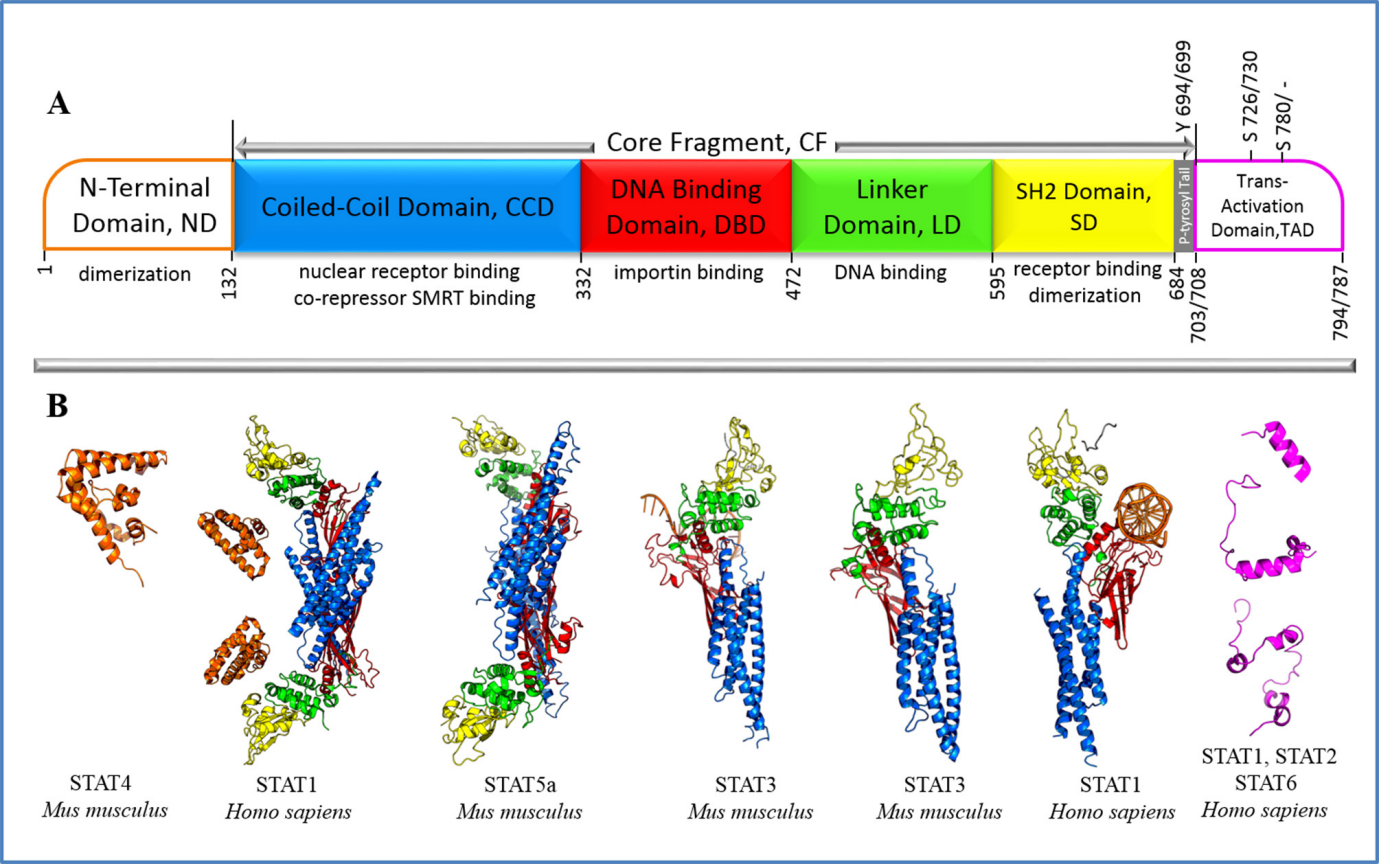
Structure of the STAT proteins. **(A)** STATs structure is composed of a N-terminal domain (N-term), a Coiled-Coil domain (CCD), a DNA Binding domain (DBD), a Linker domain (LD), a SRC homology 2 domain (SH2), a Phosphotyrosyl Tail (p-Tail), and a C-terminus named the Trans-Activation Domain (TAD); **(B)** The crystallographic or NMR data (Protein Data Bank, PDB) characterized a structure of STAT1 (1BF5 [12], 1YVL [13] and 2KA6 [14]), STAT2 (2KA4 [14]), STAT3 (1BG1 [15], 3CWG [16] and 4E68 [10]), STAT4 (1BGF [17]), STAT5a (1Y1U [18]) and STAT6 (1OJ5 [19]). Different STATs domains are distinguished by color: N-terminal is in orange, CCD is in blue, DBD is in red, LD is in green, SH2 is in yellow, p-Tail is in grey and TAD is in magenta.

*STATs* genes encode for sequences of comparable lengths (from 750 to 850 amino acids) characterized by a good similarity (from 52 to > 95%) for the human full-length sequences [6]. STATs proteins consist of N-terminal domain (N-term), Core Fragment (CF) composed of a Coiled-Coil domain (CCD), DNA Binding domain (DBD), Linker domain (LD), SRC homology 2 domain (SH2), as well as a phosphotyrosyl Tail (p-Tail) and a C-terminus called the Trans-Activation Domain (TAD) (Fig 1A). Comparison of the structural architecture of STATs proteins indicates a conservation of the overall domains organization and their functional role within the family. In particular, the N-terminal domain mediates tetrameric arrangement of STAT dimers bound to adjacent DNA sites [7], the coiled-coil domain is involved in nuclear import/export [8], the DBD controls the specificity of the STAT-DNA recognition, the adjacent linker domain ensures the appropriate structure of the DNA-binding motif and regulates nuclear export in resting cells, the SH2 domain triggers dimer formation and acts either as a phosphorylation-dependent switch to control reciprocal recognition of the STAT monomers [9] or may also regulate transcription through organization of unphosphorylated STAT dimers [10], the phosphotyrosyl tail bears the tyrosine phosphorylated by upstream activator(s) to promote parallel dimerization, and the C-terminal domain contributes to the recruiting of transcription proteins through specific phosphorylated or not serine residues [11]. However, subtle sequence differences in Core Fragment as well as drastic divergence in the C-term between STAT5a and STAT5b mediate their distinct physiological actions [1,4].

The physiological functions of STATs and the mechanisms that regulate their functional molecular interactions are reviewed in [20–23]. Although STAT5 functions and related post-translational modifications are not yet fully understood, it has been reported that STAT5 activation consists of a specific tyrosine-phosphorylation event that mediates formation of a parallel dimer through the reciprocal interactions between the phosphotyrosyl residue and the SH2 domain of the STAT5 monomers [24–27]. In a cellular environment, different forms of STAT5 have been observed, with a clear predominance of monomers, reported as the major cytoplasmic species, and antiparallel dimers, a second species largely presented in cells, stabilized by interactions between Core Fragment and N-terminal domain [28,29]. Parallel dimer of STAT5 associates with importin and translocates into the cell nucleus, binds a specific double-stranded DNA sequence and activates the transcription through recruitment of protein partners [30]. A particular tetrameric state of STAT5 has also been described in the cell nucleus [31]. The factors controlling oligomerization states of STAT5 –monomer, anti-parallel and parallel dimers and tetramer arrangement–remains a challenging question.

The equilibrium between activation and deactivation processes, tightly regulated by phosphatases, is displaced when oncogenic proteins phosphorylating STAT5 appear in cells. Such excessive STAT5 activation eventually promotes the development of numerous tumors whose nature is dependent on the upstream signaling pathway partners. As an instance, up-regulation of STAT5, as well as an increase of its phosphorylation rate have been identified in a variety of cancer ‒ chronic myeloid leukemia [32,33], mastocytosis [34,35] and prostate cancer [36]. The initial oncogenic event varies from one tumor to the other. However, they all share the common effect of phosphorylating STAT5 in a deregulated way, leading to a STAT5-dependent increased tumorigenesis. Recently, two somatic STAT5b mutations (N642H and Y665F) have been described in large granular lymphocytic (LGL) leukemia patients, emphasizing the role of STAT5 in cancer pathogenesis [37]. Moreover, it has been reported that STAT5 is a significant effector of hematopoiesis and is required for the maturation of numerous cell types. STAT5 contribution in non-cancer pathologies–auto-immune diseases or inflammation–has also been recently reported [38,39].

Given its involvement in various neoplastic or auto-immune diseases, STAT5 is potentially an important therapeutic target. Regarding its different oligomerization states, alternative strategies for development of inhibitors–targeting either the functional oligomers association, or the STAT5 binding with DNA or with other signaling proteins–should be carefully explored. So far, it has been reported a limited number of STAT5 inhibitors targeting the upstream activators (indirect inhibition of STAT5) [40], the STAT5 domains, SH2 [41–43] and DNA binding domain [44,45]. These inhibitors have very limited potency and low selectivity. It is therefore a great challenge to develop highly selective and specific molecules capable to control STAT5 activity.

To apply structure-based methodology, widely and successfully used for the development of therapeutic agents, a target structural characterization is a prerequisite step. In the present paper, we report the first 3D structural models of monomeric STAT5 and their detailed study by molecular dynamics (MD) simulations with the perspective to use these biologically relevant data to develop innovative inhibition concept(s). Characterization of the intrinsic molecular dynamics, denoted as the long-distance coupled motions associated with functional regulation in STAT5 proteins, was performed by two independent analytical methods. The signal propagation across STAT5 and the protein pockets described as putative binding sites for inhibitors, are the novel elements depicted in STATs and the essential factors for determining the site(s) which can contribute to selectivity/specificity.

## Results

### Structural data analysis

To the best of our knowledge, no structural data is available for the human STAT5. Ten partial STATs structures accessible from the Protein Data Bank (PDB) [46] report either human (STAT1, STAT2 and STAT6) or mouse (STAT3, STAT4 and STAT5a) proteins (Fig 1, S1 Table). The conserved N-terminal domain is characterized separately in STAT4 (1BGF) [17], and together with the Core Fragment (CF) in STAT1 (1YVL [13]). The CF is also described in STAT1 (1BF5 [12]), STAT3 (1BG1 [15], 3CWG [16] and 4E68 [10]) and STAT5a (1Y1U [18]). The crystallographic data of the C-terminal domain is presented as short polypeptide fragments of STAT1, STAT2 (2KA6, 2KA4 [14]) and STAT6 (1OJ5 [19]).

The structure of Core Fragment is very similar in all studied STATs and consists of an N-terminal large four-helix bundle (CCD), a central IgG-like domain mainly composed of β-strands (DBD), which constitutes the DNA binding area, an helical linker domain (LD), and the mixed α-helices/β-strands SH2 domain (Fig 1). In all mammalian STATs, the crucial phosphotyrosine residue is located in a coiled tail located at the C-extremity of the SH2 domain. These structural data provide a solid experimental cornerstone to generate biologically relevant models of the human monomeric STAT5 proteins in the unphosphorylated and phosphorylated states. We did not take into account the N-terminal domain, as its position relative to the CF in monomeric state remains unknown. We also excluded the poorly characterized C-terminal transactivation domain. We therefore report here the models of the Core Fragment of both STAT5 isoforms in a monomeric arrangement, as it represents the major cytoplasmic specie, thus targetable object [28].

### STAT5 structural models

The STAT5a and STAT5b sequences are strongly conserved (93%), with a length of 794 and 787 residues respectively. The Core Fragment of the two isoforms are differed by the five-residues insert (CESAT) in the p-Tail of STAT5b and several polymorphic replacements in CCD, DBD, LD and SH2 domain (S1 Fig). Nevertheless, it has been reported that these very similar proteins exhibit different biological activities [4].

**Fig 2:**
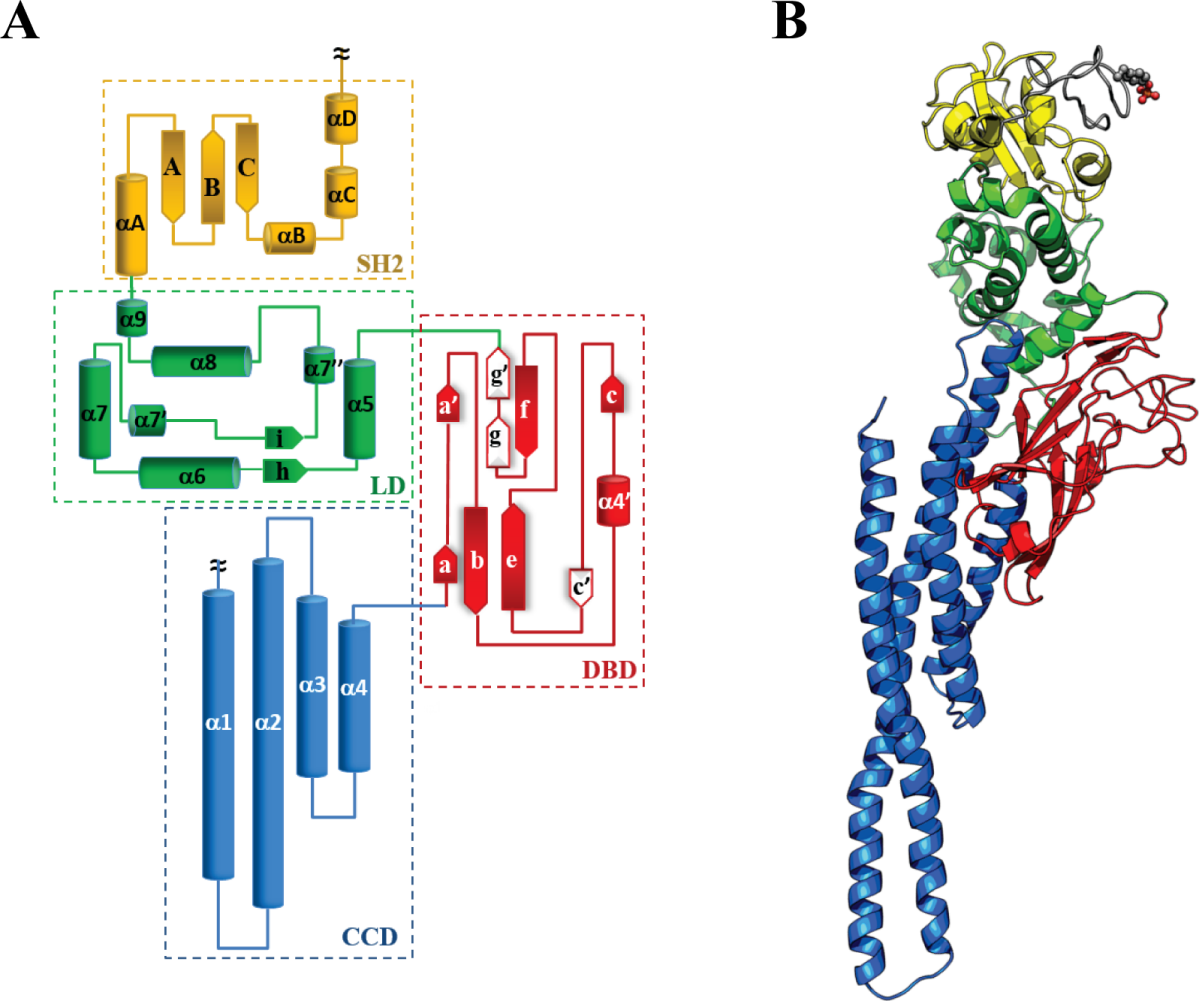
Structure of STAT5 proteins. **(A)** Topology of the STAT5 core fragment; **(B)** Ribbon diagram of STAT5 structure, the phosphotyrosine residue is shown in balls and sticks. Different protein domains are distinguished by color: CCD is in blue, DBD is in red, LD is in green, SH2 is in yellow and p-Tail is in grey.

Structural models of both isomorphs, STAT5a/STAT5b, were generated by homology from the crystallographic structures 1Y1U [18] and 1BG1 [15] in the two states, with unphosphorylated (STAT5) and phosphorylated tyrosine (p-STAT5). All STAT5 models consist of (*i*) a Coiled-Coil domain (residues 136–331), formed by four α-helices (*α1*-*α4*) packaged in a nearly parallel orientation, (*ii*) a central DNA Binding domain (residues 332–470) showing a nine-stranded β-barrel fold (denoted as *βa*, *βb*, *βe*, *βc’* and *βa’*, *βc*, *βf*, *βg*, *βg’*) and a short transient α-helix (*α4’*), (*iii*) a Linker domain (residues 471–592) formed with seven α-helices (*α5*, *α6*, *α7*, *α7’*, *α7”*, *α8* and *α9*) packed as a compact globular segment, (*iv*) a mixed α-helical (*αA-αD*) and β-sheeted (*βA-βC*) SRC homology 2 domain (residues 593–684), and (*v*) a coiled phosphotyrosyl Tail (residues 685–703 and 685–708 for STAT5a and STAT5b, respectively) with the critical tyrosine residue at position 694/699 (STAT5a/STAT5b) (Fig 2A and 2B).

As expected, the structural models of STAT5a and STAT5b exhibit a nearly perfect similarity (S2A and S2B Fig). The *α1*-*α4* helices of CCD in all STAT5 models display a comparable bending and twisting of axis (S2C Fig), providing a fine overlap between the α-helices which stabilized as an extended antiparallel coiled coil, a structural arrangement observed in all STATs. CCD packs against the two superposed β-sheets of DBD *via* a hydrophobic region coupled to a compact globular segment of LD domain formed by *α5* and *α6* helices. The DBD sheets are interconnected by extended loops which are mainly pointed outside of the domain, forming a highly flexible region, accessible to solvent, DNA or any other molecules. The loops linking the pairs of β-stands, *c* to *c’* and *e* to *f*, are particularly long (26 and 20 residues in STAT5a and STAT5b, respectively). Two helices of LD, *α8* and *α9*, produce a stable association of LD to SH2 domain through hydrophobic interactions. The coiled p-Tail, forming a compact wrapping shape, is localized at proximity of SH2. Phosphorylation of Y694/Y699 in STAT5a/STAT5b promotes a slight displacement of the coiled p-Tail from the SH2 domain towards LD in p-STAT5a and in opposite way in p-STAT5b.

### General characterization of STAT5 dynamics

**Fig 3:**
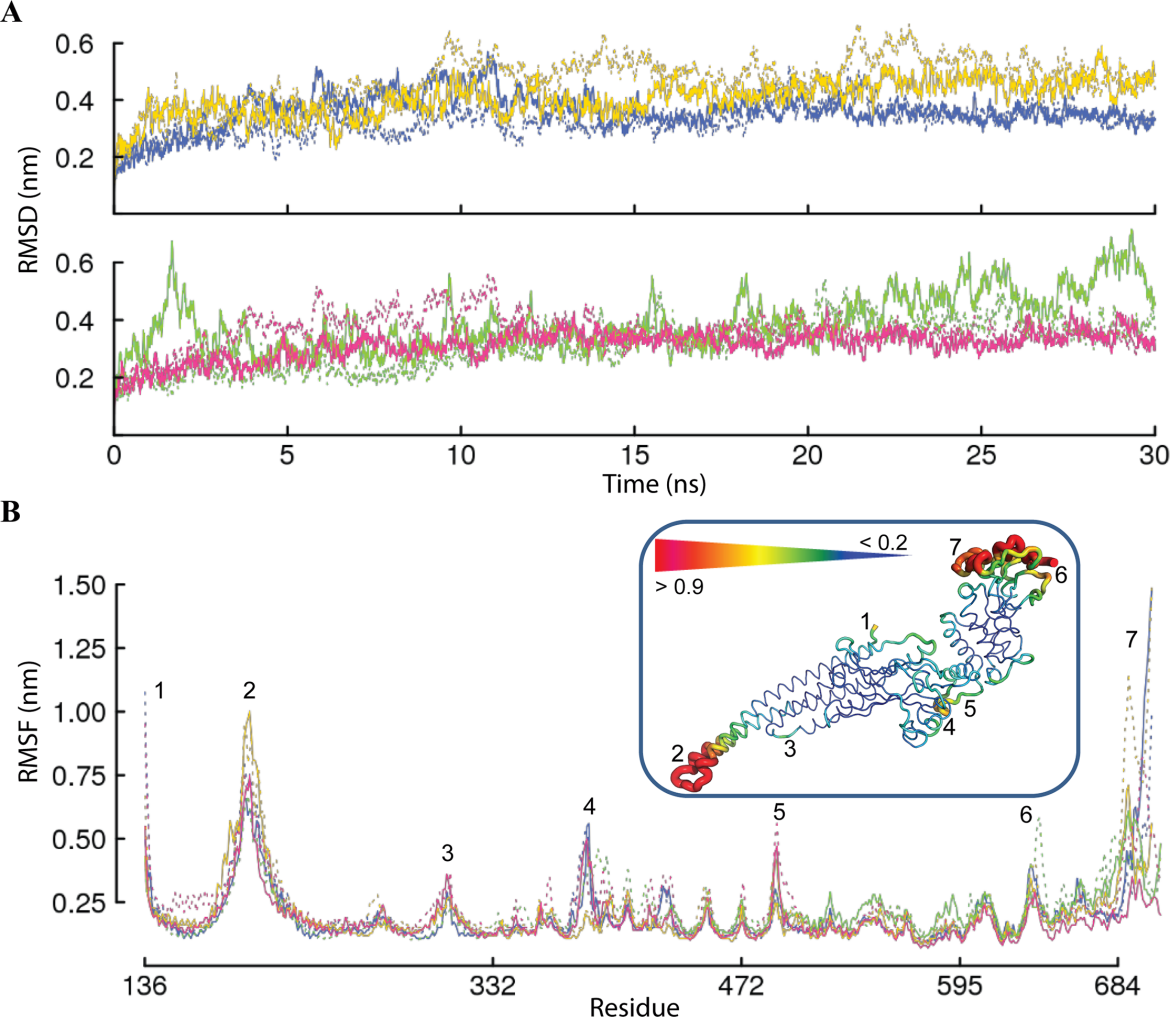
Molecular dynamics simulations of STAT5 proteins. **(A)** The root mean square deviations (RMSDs) from the initial coordinates computed on the Cα atoms for trajectories **1** (solid lines) and **2** (dashed lines) of MD simulations. The non-phosphorylated and phosphorylated (p-) states of proteins are distinguished by color: STAT5a and p-STAT5a are in blue and in yellow (top panel*)*, STAT5b and p-STAT5b are in green and in magenta, respectively (bottom panel). **(B)** The root mean square fluctuations (RMSFs) computed on the Cα atoms over the simulation time of STAT5a (STAT5a is in blue and p-STAT5a is in yellow) were compared to those of STAT5b (STAT5b is in green and p-STAT5b is in magenta). **Insert:** The average conformation for STAT5a is presented as tubes. The tube size is proportional to the by-residue atomic fluctuations computed on the Cα atoms. The high fluctuation regions are specified by color ranged from red to yellow and numbered from 1 to 7.

MD simulations (two replicas, **1** and **2)** were carried out on each STAT5 model under identical conditions. The global dynamical behavior of each simulated system was first characterized by root mean square deviations (RMSDs) computed on the Cα atoms relative to the initial structure (t = 0 ns). This analysis evidenced that (i) for each protein, the RMSD profiles of the two simulation replicas are very similar, indicating a good reproducibility of the MD simulations; (ii) a short, relaxation period, of 3–5 ns, is required to achieve a reasonable stability of the systems; (iii) the average conformational drifts are in the range of 0.30 − 0.60 nm; (iv) a good RMSD convergence is observed at the end of 30-ns simulations (Fig 3A).

For each isoform, STAT5a and STAT5b, one MD trajectory extended up to 200 ns produces the RMSD profiles similar to those observed in the 30 ns simulations (S3B Fig *versus* Fig 3A). The *per domain* analysis of the extended MD simulations showed that the CCD, DBD, LD and SH2 domains display a rapid (after 5 ns) RMSD convergence in respect to both, the initial and average structures (S4A Fig, blue, red, green and yellow curves). The tyrosyl tail, on the contrary, showed large and ample motions for the first 40 ns (STAT5a) or 50 ns (STAT5b) of simulations (S4A Fig, grey curves), which further stabilized in a steady position (S4B Fig).

The protein flexibility was estimated by the root mean square fluctuations (RMSFs) computed for the Cα atoms over the simulation time. The RMSF values range from 0.07 to 1.38 nm (Fig 3B). In all STAT5 models, the most fluctuating regions are the N-terminal residues (residues 136–140), the distal region of CCD encompassing the adjacent extremities of the α-helices *α1* and *α2* and the linker between these helices (residues 184–208), and the p-Tail (residues 684–703 in STAT5a and 684–708 in STAT5b). The significant RMSF values in the CCD distal region and especially in the loop joining two antiparallel coiled-coil helices, evidenced their great mobility. In the crystallographic structures of STAT5a (1Y1U) and STAT3 (1BG1) (the both are from *Mus musculus*), the corresponding residues display similarly high temperature (B-) factor values, although the STAT3 *α*1-*α*2 helices are significantly shorter than in STAT5. In all MD trajectories of STAT5, most of the DBD, LD and SH2 loops show increased RMSFs, associated with their high flexibility. The DBD loops linking the pair of β-strands, *c* to *c’* and *e* to *f*, are particularly exposed to solvent and their flexibility will provide them the ability to fit the DNA surface to accommodate the double-strand helix. Similarly, the p-Tail residues, highly exposed to solvent, display huge RMSF values. The RMSF profiles of the extended (200 ns) and the short (30 ns) simulations are quasi-identical (S3C Fig), displaying comparable moderate atomic fluctuations except for the C-terminal tail that shows large motions in all simulations (S4A and S4B Fig). Similarly, high RMSF values (~12 Å) have also been evidenced for the phosphotyrosyl tail of monomeric STAT3 protein [47].

### Structural features of STAT5s

Visualization and analysis of the MD conformations of all simulated models evidenced that the overall folding of Core Fragment in STAT5 is conserved and fit well to the 3D structure generated by homology. Nevertheless, the MD conformations display specific structural features for each protein, indicating first, the sequence-dependent structural properties and second, the phosphorylation-induced effects.

**Fig 4:**
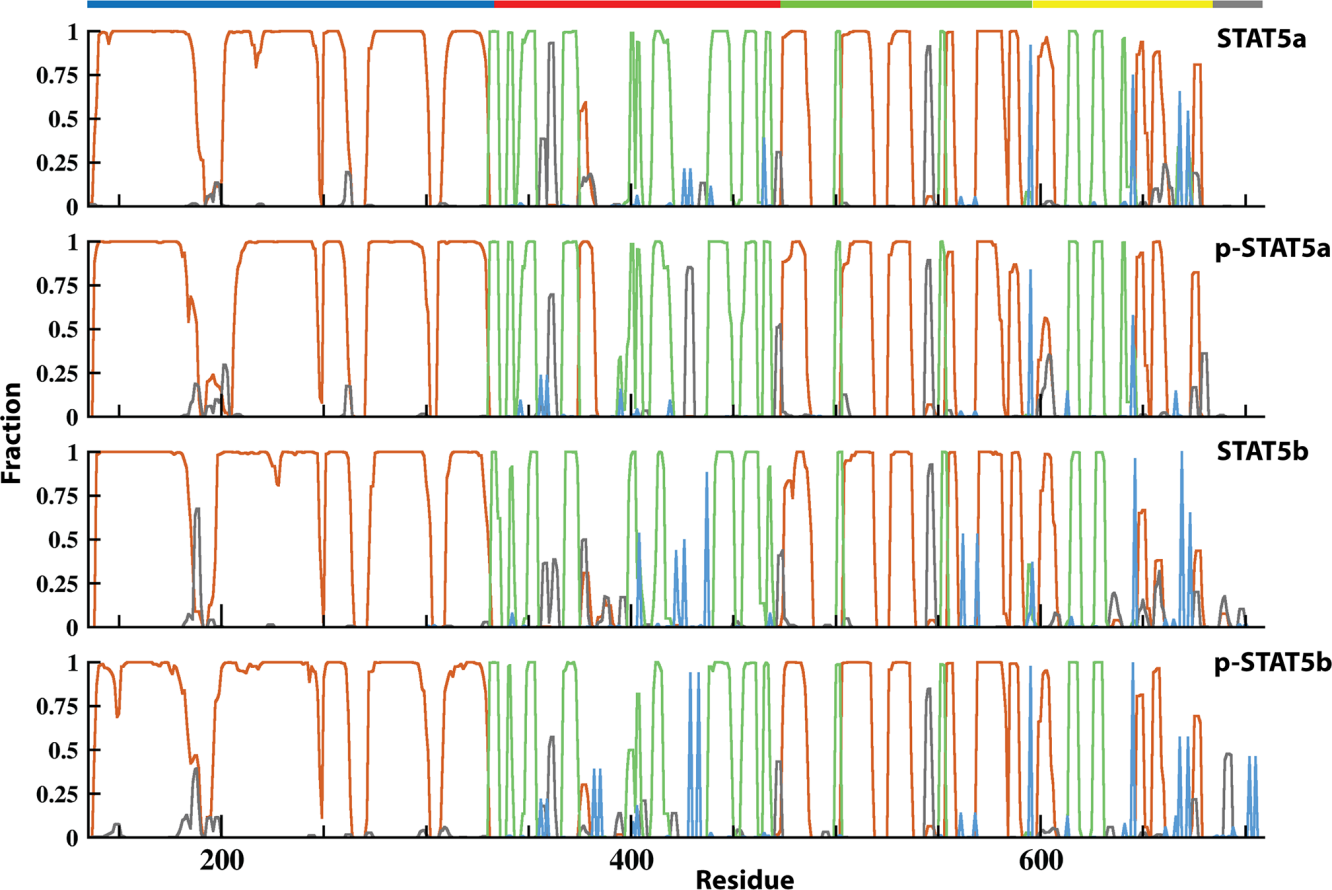
Secondary structure in STAT5 proteins. Secondary structure assignments for the STAT5 proteins were averaged over the two replicas of MD simulations. For each residue, the proportion of secondary structure type is given as a percentage of the total simulation time and shown with lines of different color: α-helix is in red, 3_10_-helix is in black, β-sheet is in green, and β-bridge is in blue. The STAT5 structural domains are indicated at the top by a colored line (the CCD in blue, the DBD in red, the LD in green, the SH2D in yellow and the C-term tail in grey).

When comparing the secondary structures evolution in STAT5a and STAT5b over MD simulations, we found that the two residues replacement in CCD, A187C and Q188F, causes a slight shift of the helical structures in STAT5b, the reorganization of *α*1-helix in C-extremity to a 3_10_-helix and the elongation of the *α2*-helix (Fig 4, S5 Fig). The point replacements in DBD (E391D, C392Y, A427S, V442I and S452C) contribute to a slight shortening of the β-strands *b* and *e*, along with a shortening of α-helix *α4’* in STA5b. We observed that the five-residues insert (CESAT) in the p-Tail of STAT5b and the series of point replacements (F636Q, N639M, L640F, K644M, S664 and F679Y) in SH2 domain promoted a significant destabilization of the helices *αB-αD* and stimulated a complete unfolding of the β-sheet C in STAT5b. We stated that these structural effects are the responses of STAT5 on the amino acids replacement (polymorphism) and consequently, may be described as a sequence-dependent structural rearrangement/adjustment having either a local character or a long-distance concerted contribution.

The phosphorylation of Y694/699 residue impacts the secondary structure of the proteins, and this effect depends on the STAT5 isoform. When regarding the secondary structure in STAT5a (Fig 4), we noticed that phosphorylation of Y694 shortens the distal region of *α1*- and *α2*-helices (CCD) and of the DBD 3_10_-helix, but in contrast, stabilizes partially the *α4’*-helix (DBD). Similarly to p-STAT5a, the phosphorylated STAT5b shows a shortening of the *α1*-helix (CCD) and a folding of the small helices (*αB-αD*) in the SH2 domain. Similarly to slight differences of the dynamical parameters (RMSDs and RMSFs) between the 30- and 200-ns MD simulations, only a tiny divergence in the secondary structures elements between short or long simulations of the same protein was observed (Fig 4, S6 Fig).

### Comparison between the STAT5a and STAT5b slow dynamics

To further explore the motions in STAT5, we analyzed the low frequency motions in STAT5, looking for the following questions: (1) Which STAT5 fragments display the larger slow motions? (2) Does the intrinsic dynamical properties are equivalent in STAT5a and STAT5b? (3) How does the phosphorylation influence the mode of motions?

**Fig 5:**
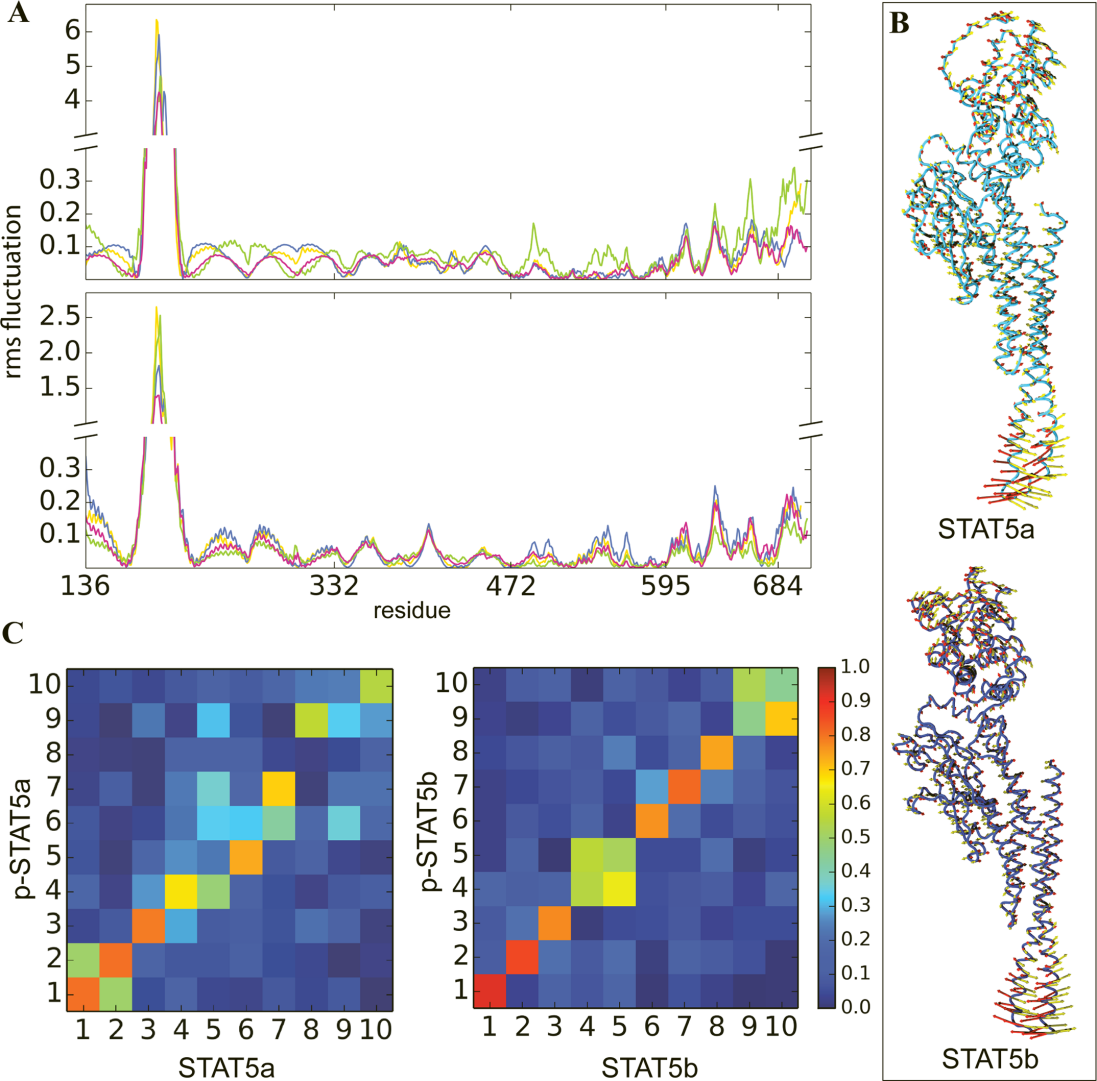
Comparison of the global dynamics by Normal Modes Analysis of STAT5 proteins. **(A)** The Cα atoms root mean square fluctuations as a function of residue number. Results for the two first modes are shown at top and bottom panels respectively, are denoted by color: STAT5a is in blue, pSTAT5a is in yellow, STAT5b is in green and p-STAT5b is in magenta. (B) First and second slowest motion modes illustrating atomic motions of STAT5a (top) and STAT5b (bottom). The STAT5 proteins displayed in cartoon representation are in light blue (STAT5a) and in dark blue (p-STAT5b). The atomic (Cα) components of each mode are drawn in red (first mode) and yellow (second mode) arrows. The length of arrows is positively correlated with motion magnitude and their orientation indicates motion direction. (C) Overlaps between the ten slowest modes of the phosphorylated and non-phosphorylated STAT5a (left) and STAT5b (right) are shown in the heatmap.

The square fluctuations of Cα atoms calculated from the first two NMA modes show that for all studied proteins, the mostly fluctuated residues are located in the distal CCD (Fig 5A). The amplitudes of the distal CCD fluctuations, explained by the first and second modes, are higher in p-STAT5a and STAT5b, than those observed in STAT5a and p-STAT5b. Other slow motions are observed in the p-Tail and in the solvent exposed loops of the linker and SH2 domains. Similarly to CCD, the fluctuations of the p-Tail residues are increased in STAT5b and p-STAT5a. This observation correlates with the square fluctuations behavior over the MD simulations. Computed scalar products between the first ten NMA modes from each pair of proteins (phosphorylated, p-STAT5 and non-phosphorylated, STAT5) indicate a good overlap between the two ensembles (Fig 5C). Namely, the modes intrinsically accessible in STAT5 are closely maintained in p-STAT5. Nevertheless, some differences are observed: while the modes 1–3 and 6–8 are maintained with an overlap of 0.7 or above in STAT5b, in STAT5a significantly fewer global modes (1–4, 7) are conserved, along with a weaker correlation and reordering (the modes 5–6 and 8–9). To describe qualitatively the most significant movements, the two first modes from STAT5a and p-STAT5b were used for illustration of the large displacements of the distal region of the CCD and the p-Tail (Fig 5B). These regions clearly demonstrate the greatest mobility in respect to the other domains in all studied STAT5.

The NMA and PCA (data not shown) results denoted that the global dynamics of STAT5 is comparable in the two isoforms, independently of their phosphorylation state. The ability of the distal region of CCD to undergo ample movements may underline the conformational adaptability of STAT5 to bind DNA or other cellular partners such as importins.

### Collective and coupled motions alternate upon phosphorylation

A general manifestation of collective motions can be obtained from the cross-correlation of atomic fluctuations. Residues that move in the same direction are correlated, while those that move in the opposite direction are anti- (or negatively-) correlated. To gain further insight into the cross-correlations, we compared the patterns in NMA and PCA cross-correlations maps. This analysis was applied to identify the dominant long-distance coupled motions in STAT5. Generally, these long-distance coupled motions are associated with functional regulation [48,49].

**Fig 6:**
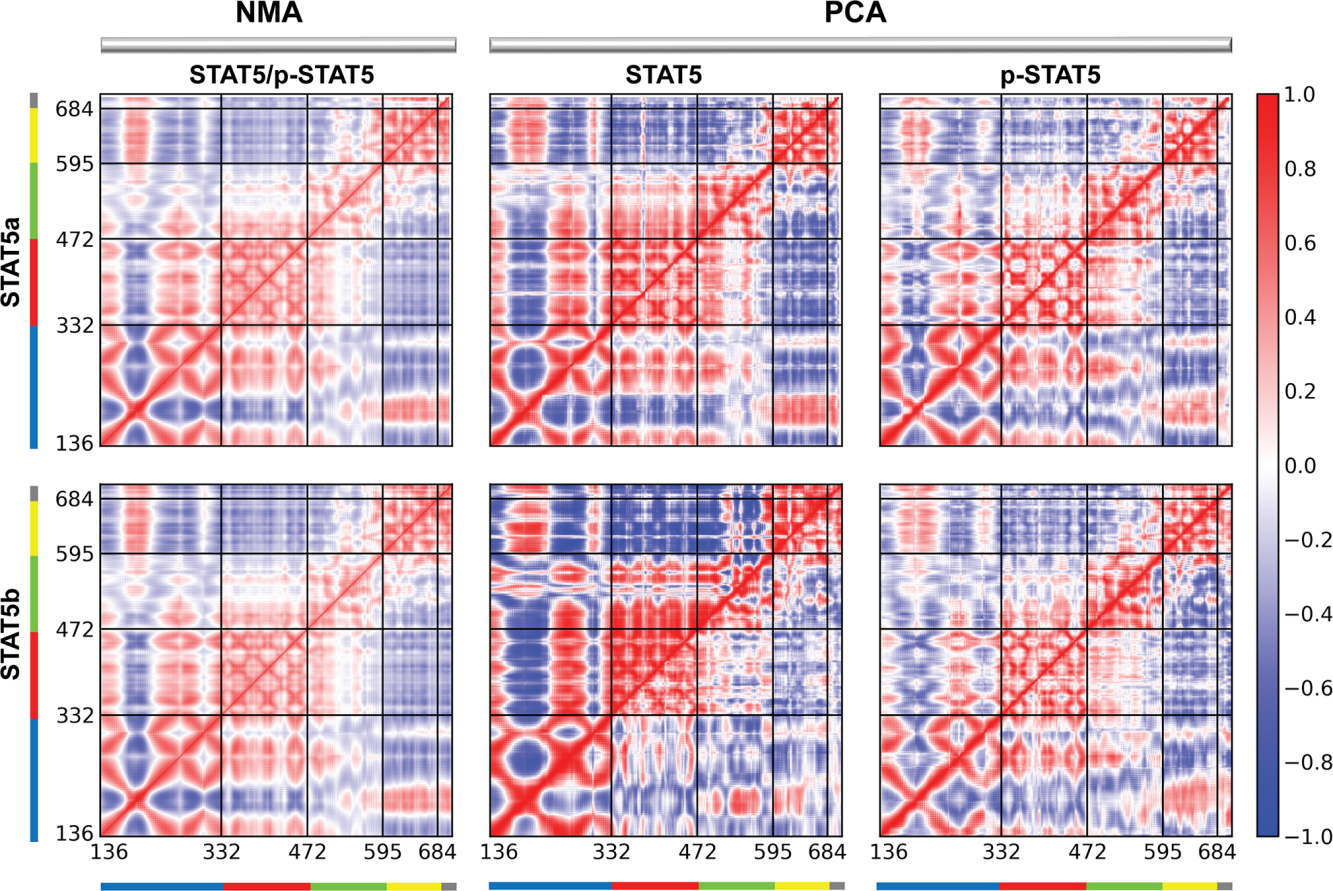
Correlated motions in STAT5. Inter-residue cross-correlations maps resulting from NMA (left column) of STAT5a/p-STAT5a (top) and STAT5b/p-STAT5b (bottom). Each protein is presented in the lower and upper half-matrix, respectively. Dynamical cross-correlations for the Cα atom pairs of STAT5a, STAT5b, p-STAT5a and p-STAT5b obtained from two MD trajectories (middle and right columns). Each replica, 1 and 2, is presented in the lower and upper half-matrix, respectively. Correlated (positive) and anti-correlated (negative) motions between atom pairs are presented as color gradient of red and blue, respectively.

The cross-correlation maps (Fig 6) were calculated with using all NMA (left column) or PCA (middle and right columns) modes. The NMA cross-correlation maps indicates that in both proteins, STAT5 (upper half of the maps) and p-STAT5 (lower half of the maps), the cross-correlation patterns are similar and indicate a highly coupled motions between largely distant sites, in particular, between the distal region of CCD (*i*.*e*., the C-extremity of *α1* and N-extremity of *α2* as well as the loop linking *α1* to *α2*, corresponding to residues 184–208) and the SH2 domain (residues 595–684), separated by 80–100 Å. The distal CCD and the N-extremity of *α1* helix, the C-extremity of *α2* and *α3*-*α4* helices (called the proximal CCD), the DBD and linker domains showed anti-correlated motions, indicating that they move in a coordinated fashion. On the other side, the distal CCD demonstrates correlated long-range motion with the SH2 domain and with the p-Tail. The strongly concerted motion of α-helices within the proximal CCD anti-correlates with the movement in SH2. Moreover, the movements in DBD and SH2 are also strongly anti-correlated. Such correlation patterns may be explained by the overall architectural features of STAT proteins, which have strongly extended (tower-like) shape. The motions of one extremity (the distal CCD) in STAT5 are counterbalanced by the motions of the opposite extremity (the SH2 domain and p-Tail) to offer a stable equilibrium to the protein around its center of gravity. Such balance should be allosterically regulated. The PCA cross-correlations computed for each MD trajectory individually show the similar patterns across all studied STAT5 and correspond to the NMA cross-correlation maps (Fig 6). The main features of dynamics correlation appeared from PCA of the non-phosphorylated STAT5s are manifested, first, by a size of the highly-correlated fragments, lengthened in the CCD, DBD and LD and shortened in SH2, and second, by an increase of the correlation values compared to the NMA cross-correlations.

A comparative analysis of the conformational mobility of STAT5 and p-STAT5 demonstrated they nearly identical structural integrity and similar motions. In details, we observed that, upon phosphorylation, the correlations and anti-correlations of all domains motion in both STAT5s are slightly diminished as evidenced by a globally weaker degree of correlations and a size variation of the correlated/anti-correlated fragments.

### Alteration of the inter-domains communication in STAT5

When characterizing the STAT5 MD conformations and their secondary structure composition, we observed some structural features specific to each protein. We hypothesized that the STAT5 folding might be a sequence-dependent and could furthermore be influenced by the phosphorylation event. The structural modifications induced by the residues replacement (polymorphism) are manifested either as a restricted folding/unfolding of neighbor fragments (local effect), or a long-distance concerted contribution observed in domains distant from the residue change (long-range effect). The phosphate transfer to the tyrosine, appear to induce the both types of effects, local and long-distance. The calculated cross-correlations (PCA and NMA) demonstrated highly coupled motions between largely distant fragments of STAT5. To understand the origin of the observed structural effects arising from different protein sequences and/or phosphorylation state, we characterized the local dynamical features of all studied STAT5s, and the intra-protein communication pathways, searching for interaction network linking the spatially distant fragments. To examine these characteristics/properties, we used the MOdular NETwork Analysis (MONETA), a method which was successfully applied to study of the allosteric communication in the receptors tyrosine kinases [50–53] and the Principal Feature Decomposition (PFD), a novel statistical approach that we developed recently.

#### Identification of the independent dynamics segments

As a first step of protein characterization with MONETA, the regions of STAT5 representing the most striking features of the protein internal dynamics were identified in each analyzed protein by a statistical technique known as Local Feature Analysis (LFA) [54], adapted to study of essential dynamics in proteins [55]. This formalism permits to identify *seed* residues in protein and further to define the clusters composed of residues neighbor to each *seed* and showing concerted local atomic fluctuations. These clusters, named *Independent Dynamic Segments* (*IDSs*), represent the protein fragments displaying independent dynamic behavior.

**Fig 7:**
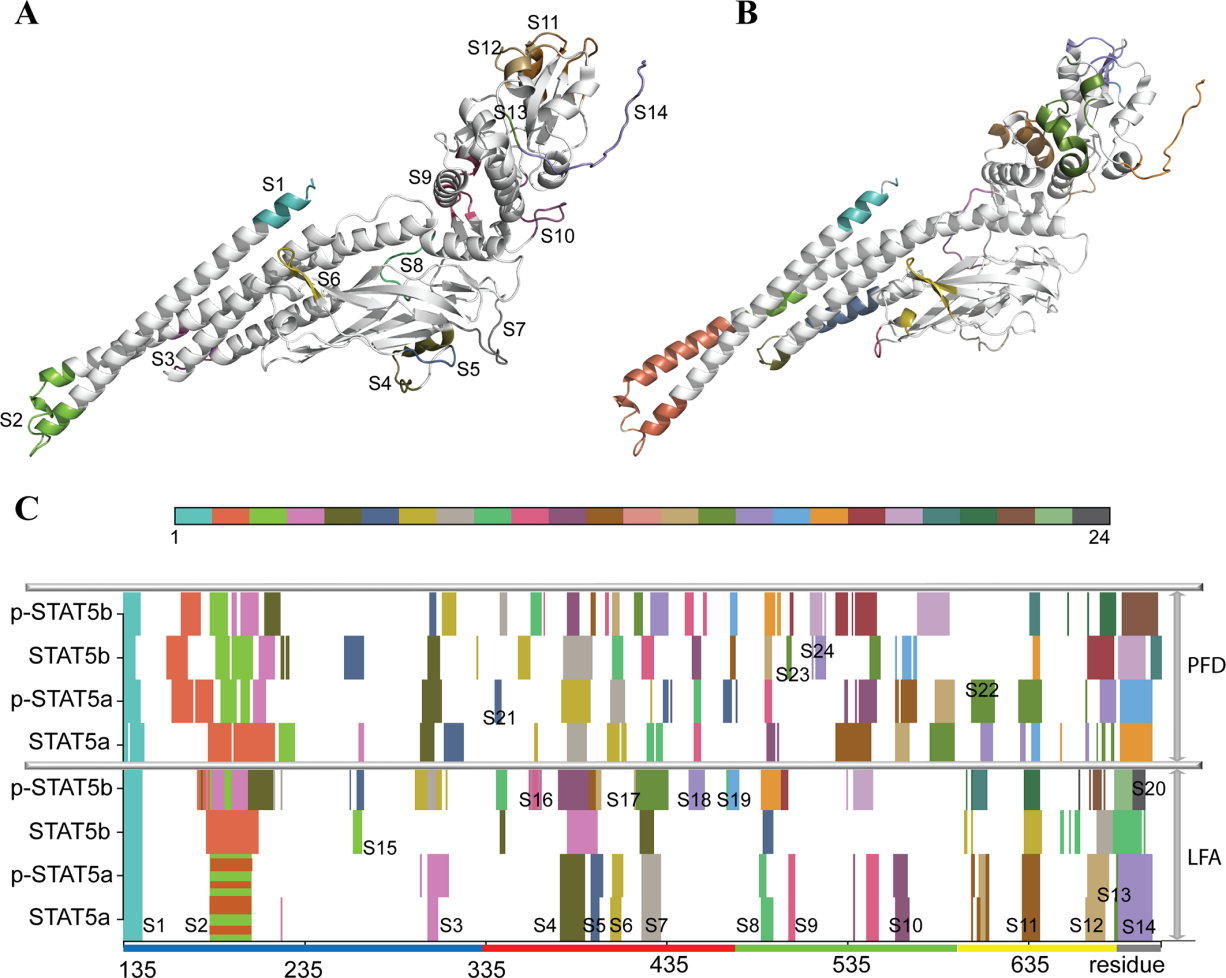
Independent Dynamic Fragments identified in STAT5 proteins. Top: 3D structural mapping of the *Independent Dynamic Fragments* (*IDSs*) in STAT5a referred to as S_i_, where i = 1, 2 …N, is presented on the average conformation as they were found by LFA **(A)** and by PFD **(B)** algorithms. Bottom: **(C)** Graph representation of *IDSs* found by PFD and LFA in each studied STAT5. Each color specifies an *IDS* obtained from a *seed* (LFA) or a *predictor* (PFD); the *IDSs* localized on the same structural fragment in various STAT5s may be colored differently according to a number of the first predicted residue in a given *IDS*.

In phosphorylated and non-phosphorylated STAT5a proteins, the number of *IDSs* was identical (14) and their positions are well-superimposed (Fig 7C). For a comparative analysis of the *IDSs* in all STAT5 models, they are referred to as S_i_, where i = 1, 2…N. Ten *IDSs* in STAT5a have discrete character (*i*.*e*., S1 or S4), the four others show partial overlapping (*i*.*e*., S2 or S11), which may be interpreted as fused or duplicated *IDSs*. The number of *IDSs* in STAT5b proteins and their character display a strong difference: 9 identified *IDSs* in STAT5b are distinct and well-separated, while 24 *IDSs* in p-STAT5b are partially overlapping and may be interpreted as 13 fused *IDSs*.

In STAT5a, the 14 identified *IDSs* are distributed over all structural domains in a nearly equal proportion (Fig 7A and 7C). They are observed mainly on the flexible regions, encompassing partially the rigid fragments adjacent to these regions. In the coiled coil CDD, three *IDSs* are located in the N-extremity of *α1*-helix (S1); in the loop linking *α1*- and *α2*-helices and in the helices portions close to the loop (S2); in the loop linking *α3*- and *α4*- helices and in the N-extremity of *α4*-helix (S3). In the DNA Binding domain, the four *IDSs* are formed by residues from the loop following the β-sheet *c* (S4 and S5), the loop connecting β-sheets *c’* and *e* (S6), and the loop linking β-sheets *e* and *f* (S7). The three *IDSs* localized in the Linker domain cover the C-extremity of *α5*-helix and extends to the adjacent loop (S8); the loop between β-sheet *h* and helix *α6* as well as the helix *α7’*, the loop linking helix *α7’* and β-sheet *i* (S9); and the loop connecting helices *α7”* and *α8* (S10). The two overlapping *IDSs* are located in the SH2 domain: S11, involving residues from the C-extremity of *αA*-helix and the loops linking the *αA*-helix to the β-sheet *A* and the β-sheets *B* to *C*, is partially intersected with S12, covering residues of the loop linking *αA*-helix and β-sheet *A*, the end of loop connecting the small helices *αC* and *αD* and the N-extremity of *αD* helix. The two last *IDSs*, S13 and S14, superimpose almost perfectly and cover the p-Tail.

In STAT5b, almost all identified *IDSs* (S1-S2, S4, S7-S8, and S12-S14), or eight from the nine, correspond well to those observed in STAT5a (Fig 7C). The five STAT5a *IDSs* (S3, S5-S6, S9 and S10) were not presented in STAT5b, while a new short *IDSs* (S15) covering residues 263–267 of CCD (the loop between *α2*- and *α3*-helices) was identified. Finally, S7 is extended to the loop between β-sheet *a’* and *b*. The *IDSs* localized in p-STAT5b are mainly superposed with those detected in STAT5a (S1-S4, S8-S9, and S11-S14) or in STAT5b (S15) or split into 2 individual *IDSs* (extended S7 *IDS* is split into S17 and S7 in STAT5b). Three *IDSs*, in CCD and DBD, were newly identified in p-STAT5b (denoted as S16, S18 and S19). The majority of found *IDSs* in p-STAT5b is overlapped and fused.

We then explored the *IDSs* in STAT5 by using the Principal Feature Decomposition (PFD), a novel statistical method developed recently to analyze protein dynamics (**Materials and Methods**). This method localized the majority of *IDSs* identified by LFA in STAT5, in particular S1-S4, S7-S8 and S11-S14 (Fig 7B and 7C). Remarkably, the large S2 distinguished by LFA at the distal CCD domain was interpreted by PFD as two (in STAT5a), three (in p-STAT5a) or four (in STAT5b and p-STAT5b) discrete *IDSs*, positioned on the coil and helices. In contrast to the spliced and superposed LFA-detected *IDSs*, the PFD-predicted *IDSs* are well-localized and have a discrete character in each studied protein, suggesting that each residue is only attached to its best *predictor*. The PFD-predicted *IDSs* are not overlapping unlike LFA-detected ones.

When comparing the results obtained by two independent methods, LFA and PFD, we observed that (i) they describe similarly the local dynamics in the studied systems and (ii) the PFD algorithm runs faster and provides a quantification of the cumulated variance of each *IDS*. Analysis of variance over PFD iterations indicated that the *IDS*s-related movements usually constitute a tiny part of all STAT5 motions (< 20%), whereas the global motions characterizing notably the concerted motions of the CCD domain respective to the LD and SH2 domains represents about 80% of the atomic fluctuations (S7 Fig). Remarkably, the global fluctuations predicted by PDF fully fit with the square fluctuations of Cα atoms calculated from the NMA modes (Fig 5A).

The PFD algorithm results in matching positions of the *IDSs* over all studied STAT5 proteins and, similarly to LFA, suggests common patterns of the local dynamics (Fig 7C, S8 Fig). The distal region of CCD and its N-extremity, the loops of DBD that contact DNA, the loop connecting the β-strands *B* and *C* in SH2, and the p-Tail are involved entirely or partially in *IDSs* localized in both isoforms having distinct phosphorylation states. Nevertheless, some specific features of the *IDSs* pattern in different STAT5 proteins may be associated with their sequence-related peculiarities/specificities and depend on the tyrosine status (phosphorylated or not). Sequence-related variations are exemplified by three observations: (i) the *IDS* S22 localized on the loop connecting *αA* helix and β-strand *A* (SH2 domain) is found only in STAT5a and p-STAT5a (Fig 7C); (ii) the *IDSs* covering the loop following *α6* in LD (S23) and (iii) the three residues between β-strand *h* and *α6*-helix in LD (S24) are specific to STAT5b systems. Regarding the *IDSs* in STAT5 proteins with different phosphorylation status, we found that S15 localized at the *α2-α3* loop is observed only in non-phosphorylated proteins (STAT5a and STAT5b), while S21 positioned at the β-strand *a’* is observed in both phosphorylated proteins, either as a spread (p-STAT5a) or as an individual (p-STAT5b) *IDS*.

#### Communication pathways

**Fig 8:**
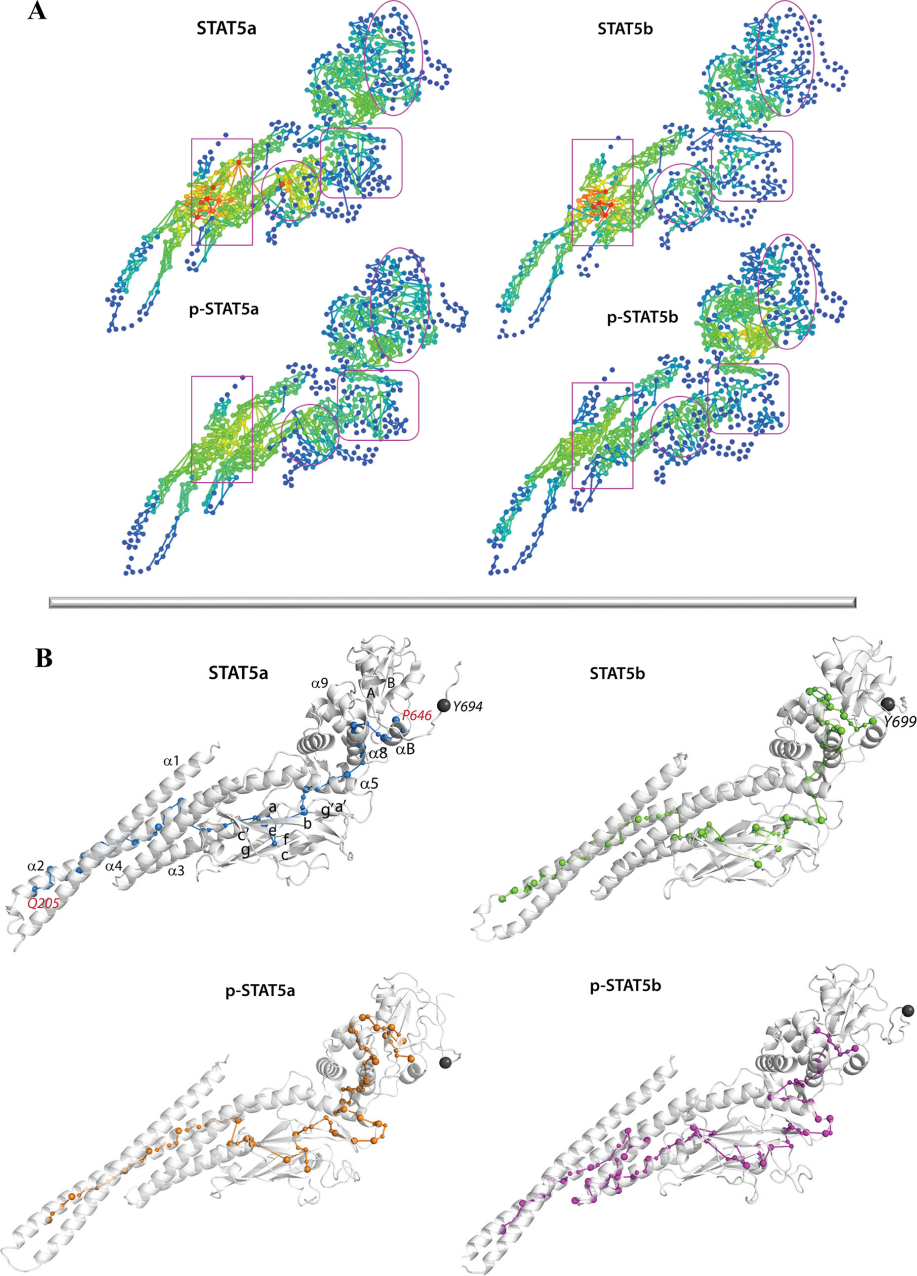
Communication in STAT5. **(A)** Global inter-residue communication represented as 2D interaction networks. Residues are presented by points, *communications pathways* are depicted by lines. Residues are colored according they *communication efficiency* (*CE*), estimated as the number of residues connected by at least on *CP*, from blue (poor *CE*) through green and yellow to red (high *CE*). **(B)** 3D structural mapping of the inter-residue communication in STAT5. For each protein, non-phosphorylated and phosphorylated, the average MD conformation is shown as a carton. Communication pathways between residues are depicted as connected tips. The STAT5 secondary structures are labeled. Specific tyrosine is denoted as a grey ball.

To analyze the communication between spatially distant regions or domains of STAT5, we computed for each model all *Communication Pathways* (*CPs*). The general landscape of *CPs* depicted as two-dimensional (2D) graphs mapping communication efficiency (Fig 8A) indicates differences in communication pattern first, between STAT5a and STAT5b and second, between non-phosphorylated and phosphorylated STAT5s.

Because the STAT5a and STAT5b sequences differ by the five-residue insert (CESAT) at the edge between SH2 domain and the p-Tail in STAT5b, and a series of point replacements in the SH2 domain, CCD, DBD and LD, we paid particularly attention to the analysis of communication pathways involving these residues and their environment. Nearly all structural domains in the *CPs* landscape of these protein isoforms show significant difference. For instance, DBD (delimited by round contour, Fig 8A) contains 5 point replacements and is characterized by a high number of *CPs* between residues of the *α5*-helix and the loop linking the β-strands *e* and *f* in STAT5a and p-STAT5a, while no such *CPs* were found in both STAT5b. Furthermore, in p-STAT5a, the *α5*-helix and the loop linking β-sheets *e* and *f* are connected by the multiple *CPs*. The second area, delineated in the SH2 domain (oval contour, Fig 8A) with the five-residue insert CESAT in STAT5b and the three others point replacements, shows a dissimilarity of communication between the β-strands *B* and *C*. Multiple *CPs* between these β-strands in STAT5a and p-STAT5a are not presented in both STAT5b proteins. Interestingly, most of the *CPs* are found between conserved residues (*i*.*e*., I629 –W631 of the β-strand B and W641 –N642 of the β-strand *C*).

When comparing phosphorylated and non-phosphorylated STAT5, we primary focused on the Y694/699 (STAT5a/STAT5b) communication paths. On the one side, this crucial tyrosine is directly engaged in *CPs* only in one proteins, p-STAT5a, making very short connections to adjacent residues of the p-Tail, while in the others proteins no *CPs* are formed by residue Y694/699 (delimited by square contour, Fig 8A). On the other side, a global analysis of the *CPs* landscape established a long-range effect of the Y694/699 phosphorylation. In particular, the CCD four-helix bundle *(α1*–*α4*) shows a dense cluster of highly connected residues in non-phosphorylated proteins, while being distant of ~50 Å from the Y694/699. In both phosphorylated STAT5, this region displays moderately connected residues. To quantify these observations, we computed (i) the number of *CPs* between each pair of helices and (ii) the number of residues connected by at least one *CP* between each pair of helices. The communication between *α1*–*α2* helices is observed in all proteins, nevertheless the number of *CPs* and amount of connected residues are clearly greater in the non-phosphorylated STAT5s (S2 Table). Similarly, the communication between *α1*–*α3* helices is considerably enhanced in the non-phosphorylated STAT5, whereas in the phosphorylated protein this communication is weak (p-STAT5a) or absent (p-STAT5b).

This detailed analysis of *CPs* showed their alternation in SH2, LD and DBD domains in the phosphorylated STAT5 in respect to the non-phosphorylated protein (Fig 8B). The “shortest” intramolecular pathway (*i*.*e*., *CP* involving a minimal number of amino acids) connecting largely distant regions in each protein joins F646, located at the C-extremity of the SH2 β-strand *C*, to Q205, positioned at the N-extremity of CCD *α2*-helix, was obtained by drawing of successive *CPs*. This generic pathway connects two spatially distant sites (Q205 and F646 are separated in space by large distance, >100 Å). The path Q205 –F646, able to transfer information from the CCD to the SH2 domain, is shorter in STAT5a compared to STAT5b and their phosphorylated species, p-STAT5a and p-STAT5b. In STAT5a, this path is 18-successive-*CPs*-long compared to 25-*CPs*-long in p-STAT5a. Surprisingly, in p-STAT5b, no connection is found between Q205 and F646 while a 30-*CPs*-long pathway is observed in STAT5b. The Q205 –F646 *communication pathway* interruption observed at the *g’*- *α5* loop stops the connection between DBD and LD in p-STAT5b. This effect illustrates how phosphate binding to tyrosine perturbs not only local fragment (p-tail) but also affects distant site(s) of the protein.

One of the striking features of this “shortest” intramolecular *communication pathway* is its circuit across SH2 domain and passage to LD. In STAT5a, the *α8*-*αB* communication is direct, while in STAT5b it involves the β-strands *A*-*B* and *α7*-helix, making a lap-like SH2 itinerary prior passing to LD. Similarly, the *CP* circuit in SH2 domain is observed in p-STAT5a. In p-STAT5b, the *CP* between SH2 and LD is neither direct nor a lap-like, and may be characterized as an intermediate route. The other marked feature of the “shortest” intramolecular *CP* is its passage from LD to DBD and CCD domains. In STAT5a proteins, this path involves a minimal number of residues, while in both STAT5b the length of the path is significantly increased through a visiting nearly all residues of the *g’-α5* loop.

### Identification of binding pockets

One of the elements that restrain the exploration of new therapeutically convincing molecules using structure-based approach is the restricted target-related data. The identification and characterization of small-molecule binding pockets are crucial factors for hit compounds search. Traditionally, the pockets search is performed on crystallographic structures or on rigid models. MD simulations can be helpful in the discovery of new binding sites, through the exploration of thousands of protein conformations describing the structural and dynamical behavior of macromolecules. The central event in STATs function is a dimerization step followed by phosphorylation of specific tyrosine residue [56]. The dimerization interface would thus represents a primary putative binding site for small molecules that may impede the phosphotyrosine binding to its target site or inhibit the conformational changes in STAT proteins necessary to the dimerization process. In STAT3 dimer, residues K591, R609, S611 and S613, located in the SH2 domain, form direct polar interactions with the phosphotyrosine pY705 [15], denoting this site as crucial for biological function(s) of STATs.

**Fig 9:**
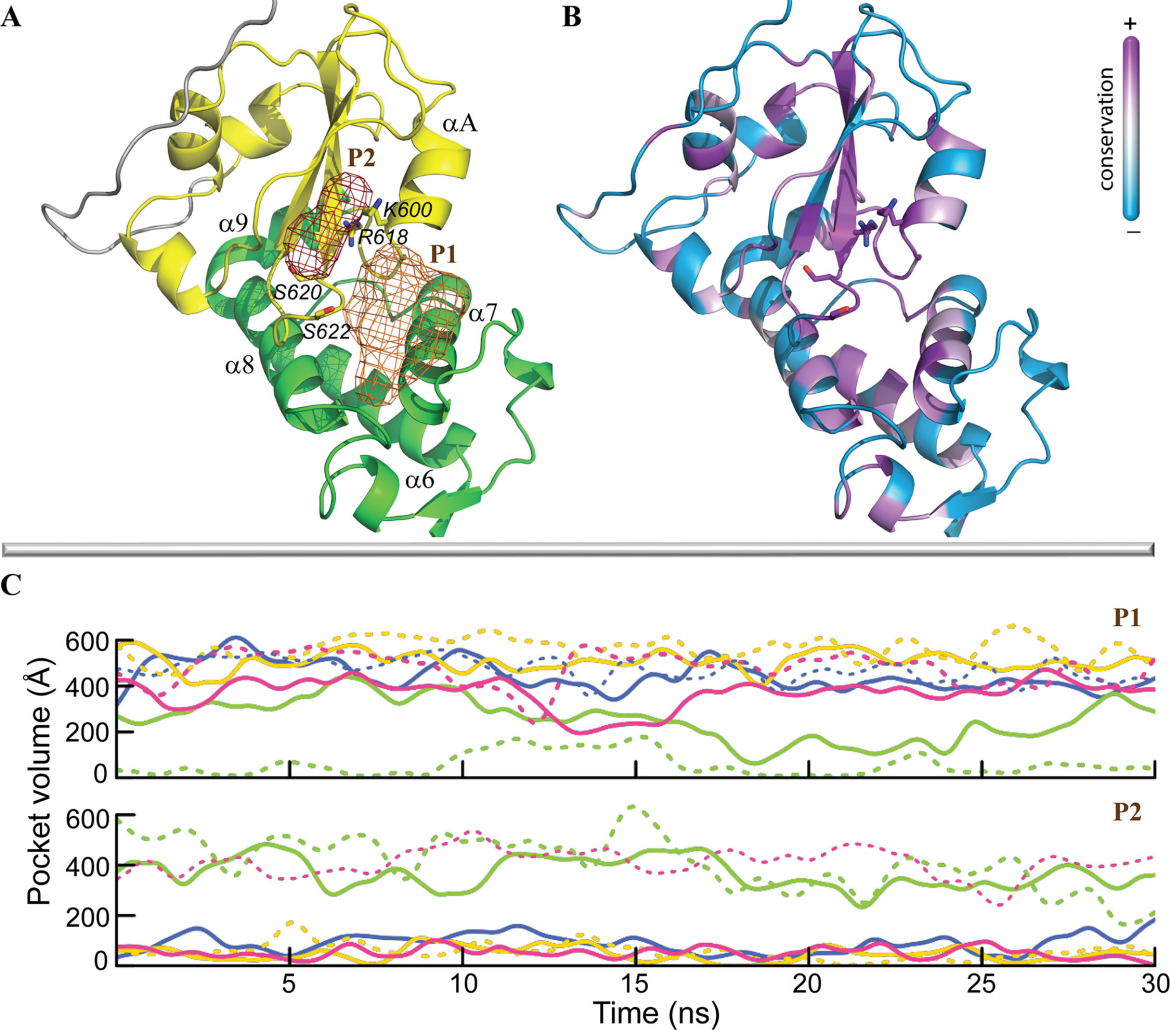
Pockets detected at the STAT5 surface. **(A)** Two pockets (brown contours) are located in LD (in green) and SH2 (in yellow) domains; the key residues, K600, R618, S620 and S622, are shown as sticks. **(B)** Sequence conservation (+)/variability (-) between STAT5 and other STAT proteins. **(C)** The pockets volume was monitored over MD simulations of each STAT5. STAT5a is in blue, pSTAT5a is in yellow, STAT5b is in green and p-STAT5b is in magenta. The two replicas, 1 and 2, for each protein are distinguished by solid and dashed lines, respectively.

The protein surface at proximity of these functionally crucial residues in STAT5 was carefully investigated with *MDpocket* [57]. We identified two adjacent pockets, P1 and P2, located between the LD and SH2 domains (Fig 9A). Pocket P1, circumscribed by helices *α*6 and *α*7, the loops linking these helices, and helix *α*A of SH2 domain, was found in all simulated proteins. The second pocket, P2, found in the SH2 domain between α-helix *A* and β-strands *A-B*, was also systematically observed, excepted in the second replica of non-phosphorylated STAT5a. These two pockets are separated by residues K600, R618, S620 and S622. Analysis of the residual conservation in proteins of STAT family indicates that residues at proximity to P2 are perfectly conserved while many residues formed P1 show the lower conservation level across STATs. (Fig 9B). Pocket P2 corresponds to the phosphotyrosine binding sub-pocket (pY+0) reported by Gianti *et al*. [58], and is targeted by most of the current STAT5-interacting molecules [41]. This site, common in all STAT proteins, contributes to formation of the parallel functional dimers, which are well characterized by X-ray crystallography for STAT1 and STAT3 [12,15]. Significantly, the pocket P1 is clearly distinct from (pY+3) sub-pocket [58] or other described pockets in STATs, and, to the best of our knowledge, represents a novel putative ligand-binding site.

To characterize the size of localized pockets P1 and P2, a second *MDpocket* run was performed. The volume of each pockets, notably alternating along the simulation time (Fig 9C), reveals the dynamic behavior of the pockets form and their dimension. The STAT5a pockets volume indicates that P1 is a bulky (~500 Å^3^) in both forms, p-STAT5a (in yellow) and STAT5a (in blue), while P2 is tiny, closed or equal to zero. In STAT5b, the pockets profiles vary differently in the two MD replicas. The size of P1 in the non-phosphorylated form (in green) varies from 0 to 200 Å^3^ and from 100 to 400 Å^3^ over the first and the second replica respectively, whereas the P2 volume fluctuates in the range of 300–600 Å^3^. In the phosphorylated STAT5b (in magenta), the pocket P1 is large, similarly to STAT5a, and its variations agree well over the two replicas, whereas the P2 size is close to zero in the first simulation and varies from 300 to 550 Å^3^ in the second one. This analysis evidenced that the two adjacent pockets, P1 and P2, located between LD and SH2 domains in STAT5, show the different profiles of their volume over the simulation of STAT5 proteins–systematically a bulky P1 and a tiny P2 in STAT5a, while in STAT5b their profiles are strongly divergent over the two simulations of the same species.

## Discussion

Numerous experimental and theoretical studies have conclusively demonstrated that structural fluctuations of proteins are often intimately coupled to biological function [59]. The key of this coupling is associated with the fundamental property of protein to alternate conformations to be able to preferentially bind different partners at distinct times and/or locations. One of the most prominent cellular objects that regulate cell signaling is STAT5, a key member of the signal transducer and activator of transcription (STAT) protein family. STAT5s, activated by a wide variety of cytokines and growth factors [60], are crucial regulators in controlling physiological cell processes (*i*.*e*., survival and proliferation of hematopoietic cells). Deregulation of STAT5 functions leads to pathological events and has been linked to a variety of human diseases. In particular, it was reported that some tyrosine kinases exert their oncogenic function by recruiting STAT5 directly in leukemic cells of human chronic myeloid leukemia, and acute lymphoid leukemia patients or mastocytosis [33,61].

We applied different computational (*in silico*) techniques to study the dynamical behavior of both isoforms of STAT5 –STAT5a and STAT5b –in non-phosphorylated and phosphorylated states. Comparative analysis of general characteristics (RMSDs, RMSFs) derived from simulations indicates that a relatively short (~10–30 ns) duration of MD simulations was sufficient to reach a good convergence and stability of STAT5 models. Further exploration of extended time-scales dynamics generating the statistically significant data is on-going but is not absolutely required for the purposes defined in this paper.

The first question was: how similar or divergent are the structural and dynamical features of STAT5 isoforms? We observed that the high sequence homology between two proteins is perfectly reflected in their structures similarity. Nevertheless, detailed analysis of the secondary structure elements in STAT5a and STAT5b over MD simulations, indicates a slight but noticeable difference between these proteins, reflecting a sequence-dependent structural arrangement. The most important structural divergence is observed in SH2 domain, the less conserved region of the Core Fragment. Functionally, SH2 domain, alone its involvement in STAT5 dimerization, is predominantly associated with the protein tyrosine kinases (PTK) signaling pathways because it specifically recognizes phosphotyrosine (p-Y)-containing motifs within the target proteins. The SH2 domain recruits peculiar proteins through the binding to specific phosphotyrosine residues, assembles multi-protein signaling complexes, and regulates protein activities [62]. Distinct physiological actions of STAT5a and STAT5b [1] may be mediated either by drastic divergence of the C-terminal domain, absent in our study, or interfered with a sequence difference in Core Fragment, in particular, in p-Tail. It has been suggested that phosphorylation sites may be affected by mutation either the phosphate-binding residue or the neighbor residues that are recognized by regulatory kinase or phosphatase [63].

STAT5 proteins exhibit a large range of internal motions, from individual atomic displacement to collective large-scale movements. The global dynamical behavior of STAT5s manifested as collective motions reflecting the functionally-related movements is comparable in both isoforms. We demonstrated that the remote side of CCD, composed of the distal portions of two extended α-helices *α1* and *α2*, displays an astonishing ability to oscillate in different directions, while the proximal segment of CCD (the four-helix bundle composed of the proximal portions of *α1*- and *α2*-helices, and of *α3*- and *α4*-helices) displays reduced motions. In the other STAT proteins (not STAT5), this dynamical feature has not been described in literature and probably is not present, due to shorter *α1*- and *α2*-helices.

Interestingly, no residues of the distal CCD has been described to be crucial for STAT5 functions–nuclear import, DNA binding or oligomerization. Consequently, the CCD motions observed in STAT5 monomers may rather reveal a sequence-encoded dynamical feature whose function needs to be explored. Cross-correlation analysis based on NMA and PCA, indicates a highly coupled motions between largely distant sites of proteins, in particular, between the distal region of CCD and the SH2 domain, separated by 80–100 Å. This feature suggests long-distance allosteric regulation of the conformational and dynamical processes operating in these proteins.

The second question relates to the STAT5 activation regulated by phosphorylation event. In general, the covalent binding of phosphate to proteins alters signaling paths by modulating phosphorylation-dependent protein-protein interactions and also by promoting conformational changes on the phosphorylated protein [64,65], its kinetics and dynamics [66], regulation of protein activity, stability and cellular localization [67,68].

STAT5 proteins have modular domains that recognize specific sequence motifs of the cell signaling proteins and of the DNA. As a first event, STAT5 modular structure implies the phosphorylation of a tyrosine residue located in the solvent exposed p-Tail, outside the specialized binding domains. Our *in silico* study indicates that the Y694/699 (STAT5a/STAT5b) phosphorylation does not influence major structural modification of STAT5, but induces rearrangement of the p-Tail leading to its partial folding, evidenced as an increasing of the β-bridges content in STAT5b and a stabilization of 3_10_-helices in STAT5a. This effect can be interpreted as a local (short-distance) issue, observed also in other phosphor-dependent proteins [69]. We showed that the Y694/699 (STAT5a/STAT5b) phosphorylation contributes to long-distance structural rearrangements in both proteins, as evidenced by either a diminishing stability of the folded regions (*i*.*e*., helices *α6*- and *αA*-helices in STAT5a, and β-strand *c’* in STAT5b) or an increase in folding (*i*.*e*., stabilization of a newly folded α-helix in DBD of STAT5a). Since STAT5b possess two serine phosphorylation sites in the Trans-Activation Domain (TAD) following P-tyrosyl tail, and STAT5a has only one, this disparity together with their specific dynamical features, probably plays a major role in the distinct *in vivo* activity reported for the STAT5 isoforms [70].

Similarly to the non-phosphorylated STAT5, cross-correlation analysis of p-STAT5 dynamics indicates highly coupled motions between largely distant sites of proteins. The motion correlations and anti-correlations in all domains of both STAT5s, upon phosphorylation, are slightly diminished, indicating a decreasing of domains motion coupling (between the CCD and SH2) which might affect the protein binding site(s) specificity for STAT5 cellular partners.

Cell-surface or cytoplasmic receptors (*i*.*e*., receptors tyrosine kinases) and signaling proteins (*i*.*e*., STATs) are important regulators of cellular tyrosine phosphorylation and mediators of intracellular signaling. A fundamental challenge in cell signaling is to address how the structure and dynamics of the signaling proteins encode and translate information produced by an initial event from membrane to downstream messengers and to the nucleus. A signal transmission between the proteins is preceded by its transmission between distant sites within a protein. Description of the intramolecular communication is a central paradigm of protein allosteric regulation. Interaction between proteins or between ligand and protein often induces local energetic and conformational changes at the binding site that subsequently propagate through the entire protein to produce conformational, dynamical and functional changes at a distant site. Such propagated conformational transitions are critical in mediating downstream signaling events. Thus, the cooperative oxygen binding properties in hemoglobin outcome from long-distance interactions between the heme groups and can be modulated by the binding of small molecules at remote sites [71]. Similarly, agonist binding to the extracellular domain of G protein-coupled receptors transduces a signal through its transmembrane domain and induces a conformational change in the cytoplasmic side of the membrane, and consequently promotes nucleotide exchange in an associated G-protein [72]. A particularly intriguing example of long-range modulation in protein is the effects induced by oncogenic mutations, which affect the tyrosine kinase activity in receptors KIT [50,52] and CSF-1R [51], and alter their sensitivity to drugs [50].

We demonstrated a coupling of protein motions between different domains in STAT5 proteins. Using two different algorithms, LFA and PFD, we identified the regions of STAT5 representing the most striking features of the protein internal dynamics, denominated as *Independent Dynamical Fragments* (*IDSs*). The LFA-detected *IDSs*, comparable in STAT5a and p-STAT5a, indicate a common pattern of the local dynamics in these systems. In contrast, the *IDSs* in STAT5b display a great variability between the non-phosphorylated and phosphorylated species. It is worth noting that the distal CCD contains perfectly overlapping (in STAT5a, p-STAT5a and STAT5b) or partially overlapping (in p-STAT5b) *IDSs*, denoting well-conserved local motions, which anti-correlate with motions of the adjacent four-helix bundle. The SH2 domain displays several partially overlapped *IDSs*, indicating a more dissociated dynamics among the different structural elements of this domain. Nevertheless, specific features of the *IDSs* in different STAT5 proteins are distinguished and can be associated with their sequence-related peculiarities and/or with their phosphorylation status. The PFD-based *IDSs* display more proper results, in terms of *IDSs* number and their composition. In the methodological context, PFD allows to identify *IDSs* according to the normalized mean variance of the residues. Thus, it provides an effective and advantageous analytical tool for exploring protein dynamics.

Interestingly, at proximity of the *IDSs* specific for a given STAT5 isoform or a phosphorylation state, no primary sequence differences (a point residue replacement or an insert) are found, indicating long-range sequence-depending effects on local dynamics. Phosphorylation of the specific tyrosine may induce a minor change in structure but modify drastically protein functions, *i*.*e*., by producing specific binding sites. In STAT5, the phosphorylation of Y694/699 influences slightly protein structure and dynamics in a canonical approximation, while the modular representation of proteins dynamics obtained with MONETA provided explicitly specificities of non-phosphorylated and phosphorylated proteins. Several unique features of STAT5s were evidenced through the *Communication Pathways* landscape which demonstrated that phosphate binding to tyrosine changed considerably the communication properties of proteins at a long distance.

**Fig 10:**
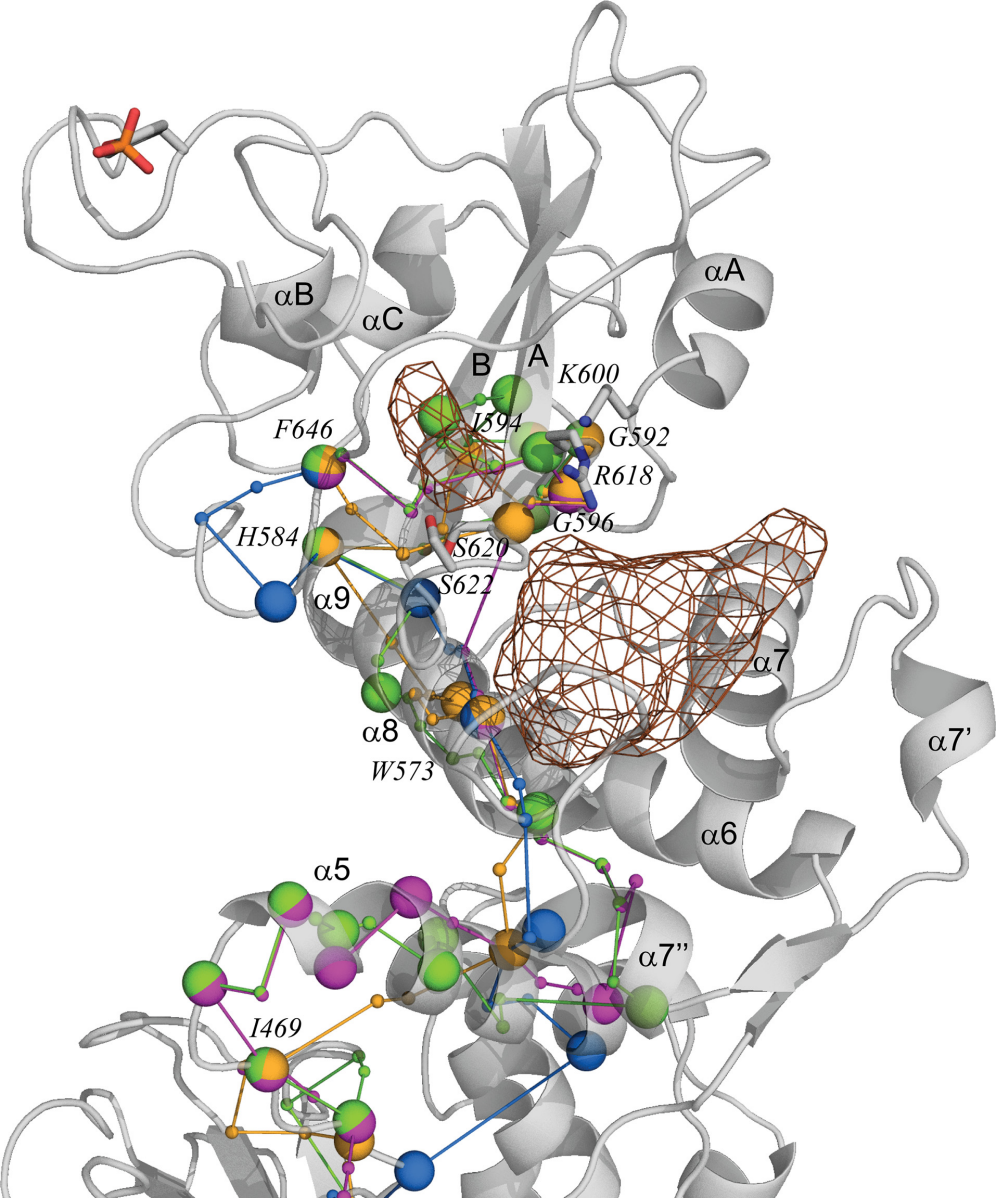
Communication pathways and pockets detected in STAT5 proteins. The Q205 –F646 “shortest” intramolecular *communication pathway* of each protein is superposed and shown together with the pockets on a carton representation of phosphorylated STAT5a. Phosphotyrosine is presented as sticks. Communication pathways between residues are depicted as connected color tips–blue in STAT5a, yellow in p-STAT5a, green in STAT5b and magenta p-STAT5b. Residues involved in more than one Q205 –F646 “shortest” intramolecular *communication pathway* are depicted as multicolor balls. The pockets P1 and P2 are shown as contoured meshes. The STAT5 secondary structures labels are shown.

To obtain physically and functionally meaningful interpretation of our findings, we superposed the Q205 –F646 “shortest” intramolecular *communication pathway* connecting the SH2 and CCD domains in each protein, together with two pockets, P1 and P2, localized in SH2-LD domains (Fig 10). Such representation evidenced that first, *communication pathways* in all studied STAT5 are localized on the same structural elements, which constitute a perfect molecular pipeline for signals transmission between spatially distant sites separated by distance more than 100 Å. Second, residue F646 participates in this Q205 –F646 “shortest” intramolecular *communication pathway* in all STAT5 and may be identified as a key residue for the STAT5 intramolecular signaling. Search of the clinically-related literature confirmed that this residue has been reported as a hot-spot point. In human STAT5b, the naturally occurring amino acid substitution F646S, the second reported mutation, is associated with severe IGF-I deficiently, immune dysfunction, and pulmonary disease [73]. Third, residue R618, participating in the “shortest” intramolecular *communication pathway* in two proteins, p-STAT5a and p-STAT5b, is one from the four strongly conserved residues in all STAT–K600, R618, S620 and S622 –defined as the borderline points between two pockets in SH2-LD domains. These residues are denoted as primordial for biological function(s) of STATs [15]. Finally, the Q205 –F646 “shortest” intramolecular *communication pathway* transiting SH2-LD domains is in closed proximity to both SH2-LD pockets.

These generic pathway features suggest a plausible using the STAT5 pockets to develop of inhibitors able to modulate communication properties of this signaling proteins. Such communication-inspired and communication-targeted modulation may block several post-transduction processes, such as dimerization, the DNA binding or upstream STAT5 activators recognition. The known inhibitors, targeting STAT5 [40–45,74,75], are active at high concentrations which are not acceptable in therapy. To design rationally the inhibitors specifically targeted STAT5, we propose to explore the communication-exposed pockets. Since the communication pattern is not conserved across the STAT5 proteins, designing the STAT5 inhibitors highly selective to a given isoform is a strategy of significant interest. Demonstration of the specific features of conformational dynamics and communication pattern in different STAT5 will also help to elucidate their role in cell signalling.

In conclusion, our study reports the structural modeling and explicit solvent molecular dynamics simulations of STAT5 proteins in monomeric form exhibiting different phosphotyrosine status. The results presented here are the first step towards the understanding of STAT5 functions. The conformational properties and peculiar features of the target dynamics were characterized by using different techniques–classical (and commonly used) (Principle Component Analysis, Normal Modes Analysis, essential dynamics, cross-correlation analysis, pocket detection) and our recently developed approaches (Modular Network Analysis and Principle Features Decomposition). The obtained results show a good coherence, indicating the data generation accuracy and the data interpretation correctness, and correspond well to experimental observations proving their biological relevance.

Two types of motions, global and local concerted motions, are described as the sequence-related and depending on phosphotyrosine status. Identification of the protein fragments representing the most striking internal dynamics and the different *communication pathway* profiles are crucial in elucidating the functional mechanisms in these allosterically regulated proteins. Reciprocal spatial position of the “shortest” intramolecular *communication pathways* and of the surface pockets in STAT5 is a significant argument to target these pockets for the development of inhibitors capable to control or modulate communication properties of these signaling proteins. Our findings will influence or change the current widely accepted paradigms into the intramolecular communication in the multi-domains STAT5, and will help to assess the physiological consequences of signaling events.

## Materials and Methods

### Homology Modeling

The protein primary sequences of the human isoforms of STAT5 (STAT5a and STAT5b) were retrieved from the NCBI protein Database (http://www.ncbi.nlm.nih.gov/protein), the NCBI reference of sequences are *NP_003143*.*2* and *NP_036580*.*2* respectively. A BLAST search of the full-length sequences against the PDB identified (i) no template available for the full-length proteins, (ii) a number of crystallographic structures of STAT as potential templates for homology modeling of the Core Fragment (CF) of STAT5 (S1 Table). The BLASTp search was subsequently restricted to residues 136–703 and 136–708 for STAT5a and STAT5b, respectively. Two structures 1Y1U [18] and 1BG1 [15] were chosen as initial templates, as they present the best sequence identity and/or the better resolution of all reported crystallographic structures of STAT, respectively. The chain A containing 138–690 residues from the unphosporylated anti-parallel dimer of STAT5a (*Mus musculus*, 1Y1U, 3.2 Å resolution), and monomeric unit A (phosphorylated) composed of 138–703 residues from the parallel β homodimer of STAT3 bound to DNA (*Mus musculus*, 1BG1, resolution of 2.3 Å) (S1 Table, S1 Fig) were used for homology modeling of the human STAT5s. The missing residues were built with Modeller 9v10 [76] and assessed using the discrete optimized protein energy (DOPE) scoring function [77]. The lowest-DOPE scored models for each phosphorylation state (phosphorylated or not) of each STAT5 isoform were retained. The quality of the homology models has been assessed by analyzing using PROCHECK [78]. Only a limited number (0.2–1.9%) of residues were found in the less favored areas of the Ramachandran plots. The ProSA-web server [79,80] has been used to compare the generated models with similar structures from the PDB; unrevealed z-scores were found in the same range than scores of the X-ray structures characterizing proteins of similar size. Finally, the MolProbity server [81] attributed scores ranging from 1.77 to 1.92 Å to our models (S9 Fig). Hydrogen atoms and the protonation state of all residues were computed using the H++ 3.0 server (http://biophysics.cs.vt.edu/H++) [82–84]. The STAT5 models were then minimized using GROMACS 4.5 [85] to remove bias geometry/interactions.

### Molecular Dynamics Simulations

#### Set up of the systems

MD simulations were performed using the models generated by homology as the initial coordinates. Set up of the systems was performed with GROMACS 4.5 [85]. First, each system comprising in about 200,000 atoms was energetically relaxed *in vacuum* using the steepest descend combined with the conjugate gradient algorithm, then solvated in a TIP3P water box and neutralized by adding counter-ions (Cl^-^), resulting in 200 000 atoms systems. Each solvated system was relaxed by 30 000 steps of minimization using the conjugate gradient algorithm. In the first 10 000 steps of minimization, constraints were applied to the protein heavy (non-hydrogen) atoms. In the following 10 000 steps, each system was minimized with constraints applied on Cα atoms only while in the last 10 000 steps, no constraints were applied. After relaxation, each system was linearly heated from 10 to 310 K with constraints on Cα. An unconstrained MD simulation was then performed at constant volume (NVT) using a Berendsen thermostat [86] for 100 ps and for further 100 ps at constant pressure (1 bar) using the Parrinello-Rahman algorithm [87]. A last MD simulation at 310 K and 1 bar (NPT ensemble) were carried out for 5 ns to achieve the properly equilibrated models.

#### Production of the trajectories

For each equilibrated system (STAT5a, p-STAT5a, STAT5b and p-STAT5b models), two independent 30-ns MD simulations (explicit solvent) were run with different initial velocities using GROMACS 4.5. The temperature and pressure were kept at 310 K and at 1 bar respectively. The LINCS algorithm [88] was applied to all bonds involving hydrogen atoms, allowing for an integration time step of 2 fs. Long-range electrostatic interactions were treated by the Particle Mesh Ewald method [89] while short-range electrostatics were cut off at 12 Å. Amber99SB*-ILDN [90–92] force field with phosphotyrosine parameters from [93] was used in all simulations. For each STAT5a and STAT5b protein, one MD trajectory was extended until 200 ns. The cosine contents of each simulation are shown in S10 and S11 Figs.

### Essential dynamics and normal modes analysis

STAT5 dynamics was characterized by using the different methods–essential dynamics (ED), and normal modes analysis (NMA). The essential directions of correlated motions during dynamics were calculated by diagonalizing the covariance matrix C*ij*, built from atomic fluctuations relative to their average positions:

$$C_{ij}=\langle \left(r_{i}-\langle r_{i}\rangle \right)\left(r_{j}-\langle r_{j}\rangle \right)\rangle$$ (1)

where *C*_*ij*_ is the element of covariance matrix, *r*_*i*_ and *r*_*j*_ are the Cartesian coordinates of atom *i* and atom *j* respectively and the brackets denote time average over the trajectory.

The average conformation was calculated over all conformations for each of the 30 ns MD trajectory (only Cα atoms were considered). The translational and rotational motions were removed by superposing each generated frame on the mean structure. The covariance matrix *Cij* was then diagonalized, producing a set of eingenvectors (or directions in a 3N-dimentional space, where N is the number of atoms) and of eigenvalues (total mean square fluctuations along the corresponding eingenvectors) [94]:

$$A^{T}CA=\lambda$$ (2)

where A represents the eigenvectors matrix and *λ* the associated eigenvalues.

The structural variations of the MD conformations were determined by *ProDy* [95] based on the concept of elastic network [96]. In this method (Anisotropic Network Model—ANM), the network nodes (*i*.*e*., Cα atoms) are connected by elastic springs with force constant γ if located within a cutoff distance of *r*_*c*_ such that the molecular potential is expressed as follows [97]:

$$V_{ANM}=\;\frac{\gamma }{2}\sum_{ij}^{M}\left(\Gamma_{ij}\right)\;{\left(\left|R_{ij}\right|-\left|R_{ij}^{0}\right|\right)}^{2},$$ (3)

where M is the number of springs, $\left|R_{ij}|-|R_{ij}^{0}\right|$ is the distance between nodes *i* and *j* with respect to the equilibrated structure, and Γ_ij_ is the element of the Kirchhoff matrix corresponding to the inter-residues contact between nodes *i* and *j*. The second derivatives of *V*_*ANM*_ gave access to the 3N x 3N Hessian matrix H, composed of super-elements (4):

$$H_{ij,j\neq i}=\frac{\gamma \Gamma_{ij}}{{\left(R_{ij}^{0}\right)}^{2}}\;\left[\begin{array}{ccc} X_{ij}X_{ij} & X_{ij}Y_{ij} & X_{ij}Z_{ij}\\ Y_{ij}X_{ij} & Y_{ij}Y_{ij} & Y_{ij}Z_{ij}\\ Z_{ij}X_{ij} & Z_{ij}Y_{ij} & Z_{ij}Z_{ij} \end{array}\right]$$ (4)

The diagonal elements of H are given by *H*_*ij*_ = −∑_*i*,*j* ≠ *i*_ *H*_*ij*_. H yields the 3N-6 non-zero eigenvalues *λ*_*k*_ and eigenvectors *u*_*k*_. The first modes associated with the lower eigenvalues (and frequencies), describe functionally relevant motions of molecules.

Calculations with *ProDy* were performed on the equilibrated pro-models (at t = 0 ns). Various spring force constants γ were applied depending on the connectivity of the nodes (*i*.*e*., γ = 10 for successive nodes, γ = 6 for a pair of nodes 7 Å apart and from the same α-helix or 6 Å apart and from the same β-sheet, finally, γ = 1 for a pair of nodes within 10 Å). Further, NMA modes computed on the homology models and the final conformations of the MD trajectories indicated a similar dynamical behavior, as evidenced by the comparable ordering of the modes (data not shown).

### Cross-Correlations Analysis

The extent to which the fluctuations of a system are correlated depends on the magnitude of the cross-correlation coefficient (CC*ij*). The CC*ij* of the atomic fluctuations obtained from the MD simulations (CC^PCA^) and the NMA (CC^NMA^) were computed using (5) [98] and (5’):

$$CC_{ij}^{NMA}=\frac{tr\left(H_{ij}^{-1}\right)}{\sqrt{tr\left(H_{ii}^{-1}\right)\cdot tr\left(H_{jj}^{-1}\right)}}$$ (5)

where *tr* is the trace of these matrices,

$${CC}_{IJ}^{PCA}=\;\frac{\langle {\Delta r}_{i}^{T}{\Delta r}_{j}\rangle }{{\langle {\Delta r}_{i}^{T}{\Delta r}_{i}\rangle }^{1/2}{\langle {\Delta r}_{j}^{T}{\Delta r}_{j}\rangle }^{1/2}}$$ (5’)

where *i* and *j* are two atoms Cα; Δ*r*_i_ and Δ*r*_j_ are displacement vectors of *i* and *j*; and Δ*r* ^*T*^ denotes the transpose of a column vector.

If *CC*(*ij*) = 1 the fluctuations of *i* and *j* are completely correlated (same phase and period), if *CC*(*ij*) = -1 the fluctuations of *i* and *j* are completely anticorrelated, and if *CC*(*ij*) = 0 the fluctuations of *i* and *j* are not correlated.

### Principal Feature Decomposition

The basic idea of Principal Feature Decomposition (PFD) is to recursively search for atoms that can predict the dynamical behavior of a subset of atoms. However, slow modes at the scale of the entire protein are an important source of correlation between distant atoms that need to be preprocessed and removed. In the sequel, we denote $X\in E=R^{3N}$ the column vector stacking the coordinates of all the *C*_*α*_ atoms, $\bar{X}=\;\langle X\rangle$ its empirical mean and $\Delta X≐X-\bar{X}$ the centered configuration. Δ*X*_*i*_ is denote the centered position of the *i* ^*th*^ *C*_*α*_ atom. The variance-covariance matrix is defined as Γ_*E*_ ≐ 〈Δ*X*Δ*X* ^*T*^〉.

#### Slow modes and canonical correlation analysis

The existence of global slow modes in the first eigenvalues of the PCA decomposition of the variance-covariance matrix Γ_*E*_ associated with eigenvectors *ψ*_*k*_ is a source of cross-correlation between potential *IDSs*. We consider the *canonical correlation analysis* (CCA) between the coordinates of two *C*_*α*_ atoms *i* and *j* by computing the canonical correlation

$$\rho_{ij}≐{\text{max}}_{u,v}\frac{\langle (u,\Delta X_{i})\left(v,\Delta X_{j}\right)\rangle }{\sqrt{\langle {\left(u,\Delta X_{i}\right)}^{2}\rangle \langle {\left(v,\Delta X_{j}\right)}^{2}\rangle }}.$$ (6)

Note that 0 ≤ *ρ*_*ij*_ ≤ 1 and that the canonical-correlation is always greater than the absolute value of the cross-correlation between *i* and *j*:

$$\rho_{ij}\geq |CC_{ij}|=\frac{\left|\langle \left(\Delta X_{i},\Delta X_{j}\right)\rangle \right|}{\sqrt{\langle {\left|\Delta X_{i}\right|}^{2}\rangle \langle {\left|\Delta X_{j}\right|}^{2}\rangle }}$$ (7)

where the right-hand side was introduced in Eq (5’). Moreover, *ρ*_*ij*_ is nothing else than the cosine of the first principle angle *α*_*ij*_ between the two 3*D* spaces generated by trajectories of the 3 coordinates of Δ*X*_*i*_ and Δ*X*_*j*_. We think that *ρ*_*ij*_ is more suitable than *CC*_*ij*_ to evaluate whether a linear dependency exists between two atoms since *CC*_*ij*_ can be vanishing even if the two vectors Δ*X*_*i*_ and Δ*X*_*j*_ are statistically strongly correlated.

On STAT5a, we found that 90% of the pair *i*, *j* for *i* ≠ *j* display a canonical correlation *ρ*_*ij*_ ≥ 0.49 and for STAT5b, the canonical correlation is even higher (0.77) (S3 Table, left; S12A Fig). In order to remove the masking effects generated by slow modes associated with the first eigenvalues, we substrate from Δ*X* its projection $\Delta X^{slow}≐\sum_{k=1}^{q}(\psi_{k},\Delta X)\psi_{k}$ on the subspace spanned by the first *q* eigenvectors (*ψ*_*k*_)_1≤*k*≤*q*_ so that the PFD procedure is started on Δ*X* ^(0)^ ≐ Δ*X* − Δ*X* ^*slow*^. The effect of the filtering is a strong decrease of the correlation *ρ*_*ij*_ when computed on Δ*X* ^(0)^ (S3 Table, right; S12B–S12D Fig). Indeed, for *q* = 6, 90% of the pairs *i* ≠ *j* have a canonical correlation below 0.64 (corresponding to a principle angle higher than 50°).

#### Principal feature Decomposition on the filtered MD trajectories

As a good compromise, we removed *q* = 6 eigensvectors before deploying the PFD on *X* ^(0)^. The first step of the PFD is an iterative selection of atoms predictors. The Residual Prediction Error (RPE) of a residue ***i*** tested as a dynamic predictor is given by the residual variance of Δ*X* ^(0)^ when optimally linearly predicted by $\Delta X_{i}^{\left(0\right)}$, *i*.*e*.:

$$\text{RPE}\left(i\right)≐{\text{min}}_{A\in M_{3N\times 3}\left(R\right)}\;\langle {∥\;\Delta X^{\left(0\right)}-A\Delta X_{i}^{\left(0\right)}\;∥}^{2}\rangle$$ (8)

where 〈 〉 is the empirical average along the concatenated trajectories of each system. The best prediction matrix *A*_*i*_ achieving RPE(*i*) is given by (9):

$$A_{i}≐\Gamma_{E,i}\Gamma_{i,i}^{-1}\;\text{where}\;\Gamma_{i,i}≐\;\langle \Delta X_{i}^{\left(0\right)}{\left(\Delta X_{i}^{\left(0\right)}\right)}^{T}\;\rangle ,\Gamma_{E,i}≐\langle \Delta X^{\left(0\right)}{\left(\Delta X_{i}^{\left(0\right)}\right)}^{T}\rangle$$ (9)

(here *a* ^*T*^ denotes the transpose of a column vector or a matrix *a*) for which

$$\text{RPE}\left(i\right)=\text{trace}\left(\Gamma_{E}-\Gamma_{E,i}\Gamma_{i,i}^{-1}\Gamma_{E,i}^{T}\right)$$ (10)

where Γ_*E*_ ≐ 〈Δ*X* ^(0)^(Δ*X* ^(0)^)^*T*^〉 is the variance-covariance matrix (in particular, Γ_*E*_ = [Γ_*E*,1_ ⋯ Γ_*E*,*N*_] is the concatenation of all the 3*N* × 3 matrices Γ_*E*,*i*_). A best predictor i_*_ ∈ *argmin*_*i*_RPE(*i*) is chosen achieving the lowest residual prediction error giving the first detected predictor *i*_1_ = i_*_. Then the optimally predicted part $\hat{\Delta X^{\left(0\right)}}≐A_{i_{*}}\Delta X_{i_{*}}^{\left(0\right)}$ is removed from Δ*X* ^(0)^ giving the residual configuration $\Delta X^{\left(1\right)}\leftarrow \Delta X^{\left(0\right)}-\hat{\Delta X^{\left(0\right)}}$ and residual variance-covariance matrix $\Gamma_{E}^{\left(1\right)}\leftarrow \Gamma_{E}-\Gamma_{E,i_{*}}\Gamma_{i_{*},i_{*}}^{-1}\Gamma_{E,i_{*}}^{T}$. The process is repeated on Δ*X* ^(1)^ giving iteratively a sequence of predictors $P=\left\{i_{1},\cdots ,i_{P}\right\}$ until a predefined number *P* of predictors is reached.

#### Condensation around atom predictors

A last part for the PFD computation is the condensation of residues around the set of predictors. For any C_α_ atom *i*, it computes the smallest normalized linear prediction error of the displacement $\Delta X_{i}^{\left(0\right)}$ among all the atoms predictors *i*_*k*_:

$$\text{VPR}\left(i\right)≐{\text{min}}_{1\leq k\leq P,A\in M_{3\times 3}\left(R\right)}\frac{\langle {\left|\Delta X_{i}^{\left(0\right)}-A\Delta X_{i_{k}}^{\left(0\right)}\right|}^{2}\rangle }{\langle {\left|\Delta X_{i}^{\left(0\right)}\right|}^{2}\rangle }.$$ (11)

The *C*_*α*_ atom is affected to its best predictor *i*_*k*_ if VPR(*i*) ≤ *r* where *r* is a threshold giving *P* non intersecting subsets *C*_1_, ⋯, *C*_*P*_. A last fusion step is done such that close by clusters *C*_*k*_ are condensed together. More precisely, an undirected graph is defined between the *C*_*k*_ taken as the set of vertices where two clusters *C*_*k*_ and $C_{k^{\prime }}$ are linked at level d_*_, noted $C_{k}↔C_{k^{\prime }}$, if $d_{k,k^{\prime }}≐min_{i\in C_{k},j\in C_{k^{\prime }}}\left|{\bar{X}}_{i}-{\bar{X}}_{j}\right|\leq \;d_{*}$. The connected components of the graph are computed and all the clusters belonging to the same connected component are fused together. In the subsequent experiments, we take d_*_ = 5Å, *q* = 6 and *r* = 0.5.

### The *Independent Dynamic Segments* identification by Local Feature Analysis

The *Independent Dynamic Segments* (*IDSs*) identification from Local Feature Analysis (LFA) [54,55] based on PCA was reported in [52]. PCA calculations were performed for all STAT5 models, on concatenated trajectories from each pair of MD replicas. From the 3N eigenvalues associated with the 3N eigenvectors, the first 8, 8, 3 and 8 eigenvectors were sufficient to describe 80% of the total Cα atomic fluctuations on STAT5a, p-STAT5a, STAT5b and p-STAT5b. These vectors were used to apply the LFA formalism as we described previously [99]. A threshold value P_cut_ was arbitrary chosen by the program to keep 1.2% of all LFA cross-correlations above it. The value was set to 0.032 for STAT5a, p-STAT5a and 0.019, 0.035 for STAT5b and p-STAT5b, respectively. Distance matrices consisting of the average of the smallest distance between each residue pairs were computed using the *g_mdmat* module of GROMACS 4.5. Two residues were considered neighbours when the average smallest distance between them was lower than a given threshold d_cut_ of 3.7 Å.

### Analysis of intramolecular communication

Modular network representations of STAT5 proteins were built and visualized with MONETA, using the latest version [99]. *Communication Pathways* (*CPs*) were generated based on the communication propensities [100] between all protein residues as was described elsewhere [53]. The *CPs* are grown in a way ensuring that any two adjacent residues are connected by non-covalent interactions and that every pair of residues in a given *CP* is connected by a short commute time (*CT*). Non-bonded interactions were recorded along the MD simulations using LIGPLOT [101]. Two residues were considered as interacting when they formed at least one non-bonded interaction during 50% of the time of simulation. To discriminate between large and short *CTs*, the threshold *CT*_*cut*_ was set to 0.1, so that highest connected residues communicate efficiently with about 6 to 9% (34 to 50 residues) of the total number of residues in the protein.

Statistical analysis of data were performed with the R software [102]; structure visualization and graphical characterization of interaction and communication are performed with PyMOL [103] using custom functions from MONETA package [99].

### Pockets detection and analysis

The proteins pockets were detected using *MDpocket* [57], a software based on the *fpocket* algorithm [104]. For each MD conformation, *MDpocket* associates the α-spheres (*i*.*e*., spheres contacting with four protein atoms without any other atom within the sphere) to a grid point. The pockets maps were produced by iterating this step over all conformers employing the default parameters. *MDpocket* requires the superposition of each conformer on a reference structure prior to any calculation to avoid artefacts which may occurred over the grid-based process. To capture the phosphotyrosyl pocket, the SH2 domain *Cα* atoms of K600, R618-E623, N642 and K/M644 (for STAT5a/STAT5b, respectively) were used to superimpose all snapshots prior to the first *MDpocket* run. Given the crucial role of the SH2 domain in the STATs functions, a first pocket detection run was performed using a superposition of the SH2 Cα-atoms. The selected pockets were further analyzed in a second *MDpocket* run. For each selected pocket, the grid points with an occupancy time of at least 0.25 (*i*.*e*., grid points associated with α-spheres at least 25% of the simulation time) were retained to extract the pocket’s metrics, such as volume, over the MD trajectory. The conservation scores were computed using the ConSurf Server [105–107].

## Supporting Information

S1 Fig Homology modelling of STAT5 proteins. Sequence alignment of the human STAT5a (accession number *NP_003143*.*2*), STAT5b (accession number *NP_036580*.*2*) and the *Mus musculus* proteins STAT5a and STAT3 from structures 1Y1U [18] and 1BG1 [15]). All aligned sequences contain amino acids 136–703 (STAT5 numbering) as was defined in the X-Ray structures. Different domains of proteins are distinguished by color: CCD is in blue, DBD is in red, LD is in green, SH2 is in yellow and p-Tail is in grey. Fully conserved (identical) residues are delineated by coloured background specified for the related structural domain; semi-conserved (similar) residues are contoured; residues showing a difference between STAT5a and STAT5b are denotes by cyan background; the conserved crucial phosphotyrosine is indicated by a red star.
(TIF)

S2 Fig The STAT5 models generated by homology. Superimposed models of STAT5a (A) and STAT5b (B) in non-phosphorylated (in blue and in green, respectively) and phosphorylated (in yellow and in magenta, respectively) states. Structures of the proteins are shown in two orientations: side view (in top panel); top view (in bottom panel). Phosphotyrosine residues are shown as sticks. (C) The CCD *α1*-*α4* helices local curvature in STAT5 models denoted by color: STAT5a is in blue, pSTAT5a is in yellow, STAT5b is in green and p-STAT5b is in magenta. Curves were derived from the trajectories 1 (solid lines) and 2 (dashed lines) of MD simulations. The local curvature of crystal structure of the mouse STAT5a (PDB 1Y1U) is shown in black.
(TIF)

S3 Fig 200 ns molecular dynamics simulations of STAT5 proteins. The root mean square deviations (RMSDs) computed on the Cα atoms from the MD trajectories of STAT5a (in orange) and STAT5b (in blue) from (A) the average conformation coordinates and (B) the initial structure coordinates. (C) The root mean square fluctuations (RMSFs) computed on the Cα atoms over the total simulation time, 30 ns (in cyan and in red) and 200 ns (in blue and in orange) for STAT5a and STAT5b, respectively.
(TIF)

S4 Fig Extended MD simulations of STAT5 proteins. (A) RMSD profiles characterizing each structural domains over the extended MD simulations (200 ns) *versus* the initial conformation (t = 0 ns) (top panel) and the 200 ns time-averaged structure (bottom panel). The RMSDs of the STAT5 domains are showed by different color: the CCD in blue, the DBD in red, the LD in green, the SH2D in yellow and the C-term tail in grey, all Cα in black. (B) Displacements of the C-term along the extended MD trajectories of STAT5a (left) and STAT5b (right), colored from red (initial conformation) to blue (final conformation). For clarity, the other STAT5 domains are shown in the transparency.
(TIF)

S5 Fig Secondary structures variations in STAT5 proteins. Secondary structure assignments for the STAT5 proteins, focusing on α-helix, 3_10_-helix, β-strand and β-bridge separately. We compare the differences between the STAT5a and STAT5b isoforms (blue and yellow lines, top panels), and between phosphorylated and unphosphorylated STAT5 (dark green and salmon lines, bottom panels). The significant differences (*i*.*e*., > standard-deviation) in secondary structure of STAT5 are indicated by red arrows.
(TIF)

S6 Fig Secondary structures in STAT5 proteins. Secondary structures assignment for the STAT5 proteins over the two replicas of MD simulations over the 30- and 200-ns trajectories. For each residue, the proportion of secondary structure type is given as a percentage of the total simulation time and shown with lines of different colour: α-helix is in red, 3_10_-helix is in black, β-sheet is in green, and β-bridge is in blue.
(TIF)

S7 Fig The STAT5 dynamics characterization by Principle Features Decomposition. Atomic variance, atomic variance after global removal (q = 6) and atomic variance predicted by the best *predictor* are shown in red, in violet and in blue respectively. *Independent Dynamics Segments (IDSs)* are shown as colored sequence segments on the X-axis.
(TIF)

S8 Fig Superposition of the *IDSs* localization predicted by Principle Features Decomposition in STAT5. Each *IDS* in STAT5a, p-STAT5a, STAT5b and STAT5b is presented by a minimal covering square. The heat map represent the residual canonical correlations after removal of the q = 6 slowest PCA modes.
(TIF)

S9 Fig Quality assessment of the homology models. The ProSa-web outputs are shown for STAT5a (upper left), pSTAT5a (upper right), STAT5b (lower left) and pSTAT5b (lower right), assessing the overall model quality. The black points indicate the models compared to the PDB X-Ray (light blue) and NMR (deep blue) structures. The ProSa z-score and the model resolution as determined by MolProbity are shown in the bottom left corner of each panel.
(TIF)

S10 Fig Cosine content of the eigenvectors of the short simulations. Three windows of time simulation are used to compute the cosine content of the 10 first PCA eigenvectors: the whole simulation (0–30 ns), the last 20 ns (10–30 ns) and the last 10 ns (20–30 ns), for each simulation.
(TIF)

S11 Fig Cosine content of the eigenvectors of the extended simulations. Seven windows of simulation time are used to compute the cosine content of the 10 first PCA eigenvectors: the whole simulation (0–200 ns), the last 190 ns (10–200 ns), the last 180 ns (20–200 ns), the last 170 ns (30–200 ns), the last 150 ns (50–200 ns), the last 100 ns (100–200 ns), and the last 50 ns (150–200 ns), for each simulation.
(TIF)

S12 Fig Canonical correlation analysis for STAT5 before (A) and (B-D) after filtering. From left to right: STAT5a, p-STAT5a, STAT5b and STAT5b. From (B) to (D): number *q* of eigenvectors removed (fist row, *q* = 4; second row, q = 6; third row, q = 8). Correlated motions between Cα atom pairs are presented as color gradient of red (CC = 1) and blue (CC = 0).
(TIF)

S1 Table The STAT proteins characterized by X-ray or NMR and deposited in the Protein Data Bank (PDB). (PDF)

S2 Table The inter-residue communication characteristics between helices in CCD. The values indicate, for each pair of helices, how many pairs of residues are connected by at least one *Communication Pathway*, and the total number of *Communication Pathways* (in brackets).
(PDF)

S3 Table Canonical correlation. 1^st^ and 9^th^ 10-quantile values (first and second lines respectively) for the canonical correlation *ρ*_*ij*_ before (left) and after (right) removal of the projection on the 6 first eigenvectors (80% of the pairs *i* ≠ *j* have a correlation which is between the two values).
(PDF)
