# Supplementary figures and images for: How Intrinsic Molecular Dynamics Control Intramolecular Communication in Signal Transducers and Activators of Transcription Factor STAT5
Source: PLoS One. 2015 Dec 30;10(12):e0145142. doi: 10.1371/journal.pone.0145142 (PMC4696835; doi:10.1371/journal.pone.0145142)

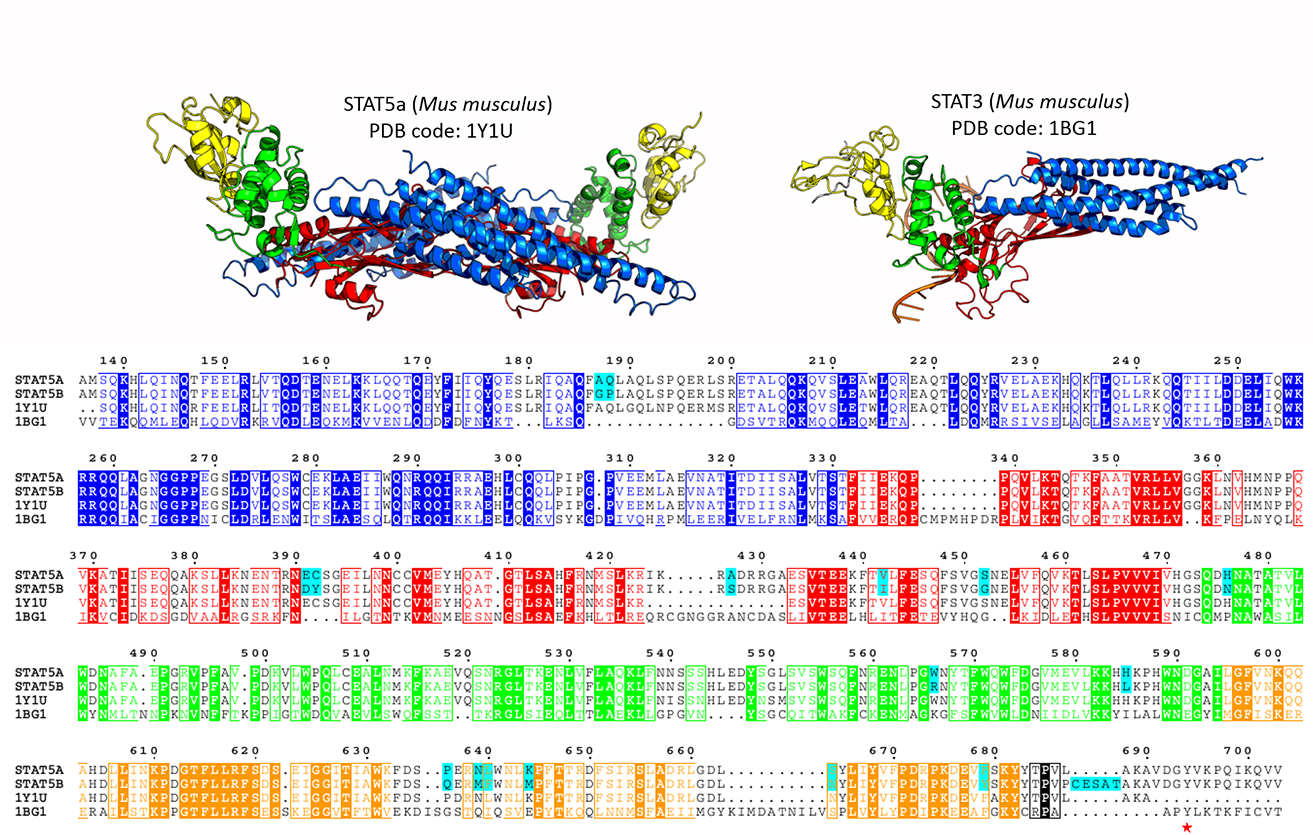

Supplement: S1 Fig — Sequence alignment of the human STAT5a (accession number NP_003143.2), STAT5b (accession number NP_036580.2) and the Mus musculus proteins STAT5a and STAT3 from structures 1Y1U [18] and 1BG1 [15]). All aligned sequences contain amino acids 136–703 (STAT5 numbering) as was defined in the X-Ray structures. Different domains of proteins are distinguished by color: CCD is in blue, DBD is in red, LD is in green, SH2 is in yellow and p-Tail is in grey. Fully conserved (identical) residues are delineated by coloured background specified for the related structural domain; semi-conserved (similar) residues are contoured; residues showing a difference between STAT5a and STAT5b are denotes by cyan background; the conserved crucial phosphotyrosine is indicated by a red star. (TIF) [file pone.0145142.s001.tif]

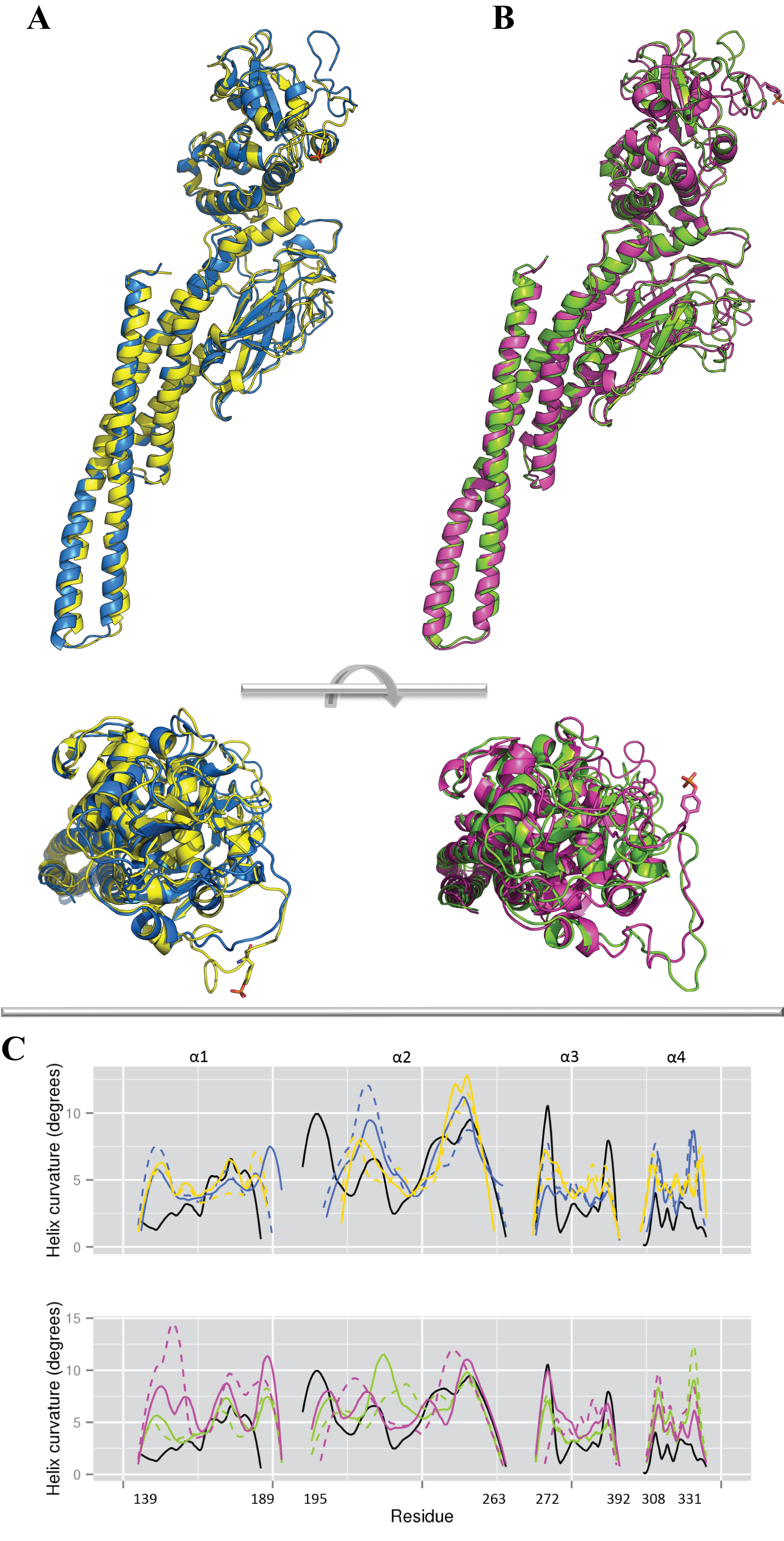

Supplement: S2 Fig — Superimposed models of STAT5a (A) and STAT5b (B) in non-phosphorylated (in blue and in green, respectively) and phosphorylated (in yellow and in magenta, respectively) states. Structures of the proteins are shown in two orientations: side view (in top panel); top view (in bottom panel). Phosphotyrosine residues are shown as sticks. (C) The CCD α1-α4 helices local curvature in STAT5 models denoted by color: STAT5a is in blue, pSTAT5a is in yellow, STAT5b is in green and p-STAT5b is in magenta. Curves were derived from the trajectories 1 (solid lines) and 2 (dashed lines) of MD simulations. The local curvature of crystal structure of the mouse STAT5a (PDB 1Y1U) is shown in black. (TIF) [file pone.0145142.s002.tif]

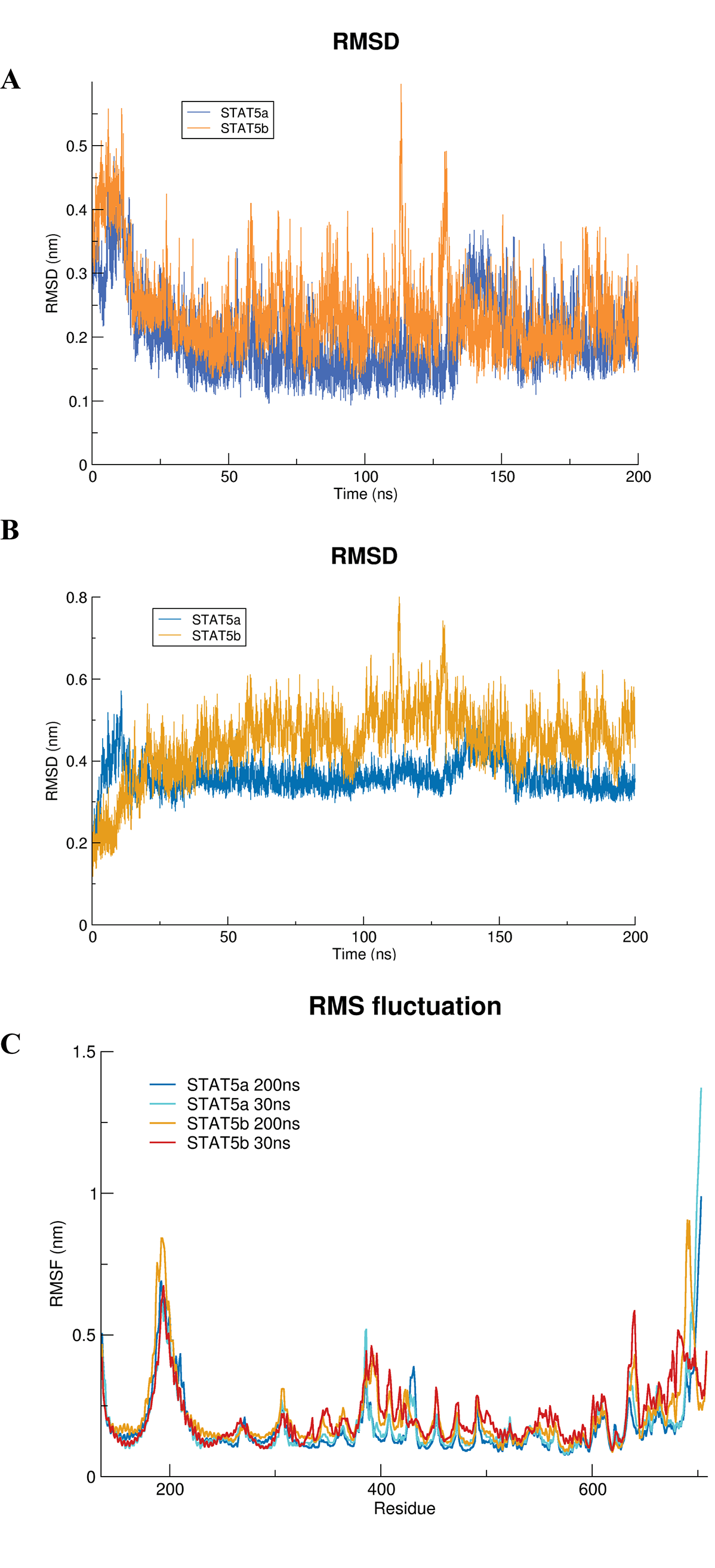

Supplement: S3 Fig — The root mean square deviations (RMSDs) computed on the Cα atoms from the MD trajectories of STAT5a (in orange) and STAT5b (in blue) from (A) the average conformation coordinates and (B) the initial structure coordinates. (C) The root mean square fluctuations (RMSFs) computed on the Cα atoms over the total simulation time, 30 ns (in cyan and in red) and 200 ns (in blue and in orange) for STAT5a and STAT5b, respectively. (TIF) [file pone.0145142.s003.tif]

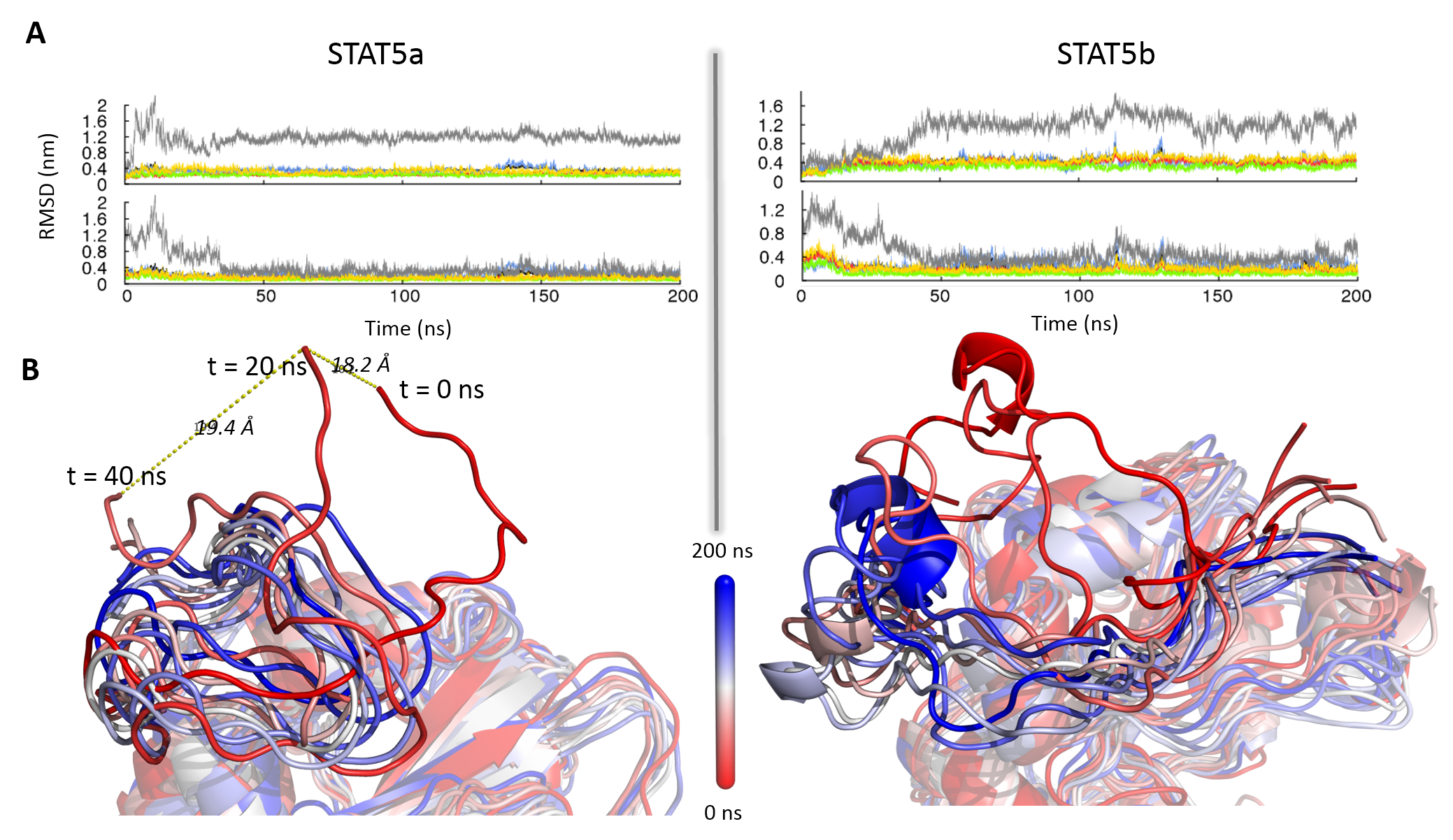

Supplement: S4 Fig — (A) RMSD profiles characterizing each structural domains over the extended MD simulations (200 ns) versus the initial conformation (t = 0 ns) (top panel) and the 200 ns time-averaged structure (bottom panel). The RMSDs of the STAT5 domains are showed by different color: the CCD in blue, the DBD in red, the LD in green, the SH2D in yellow and the C-term tail in grey, all Cα in black. (B) Displacements of the C-term along the extended MD trajectories of STAT5a (left) and STAT5b (right), colored from red (initial conformation) to blue (final conformation). For clarity, the other STAT5 domains are shown in the transparency. (TIF) [file pone.0145142.s004.tif]

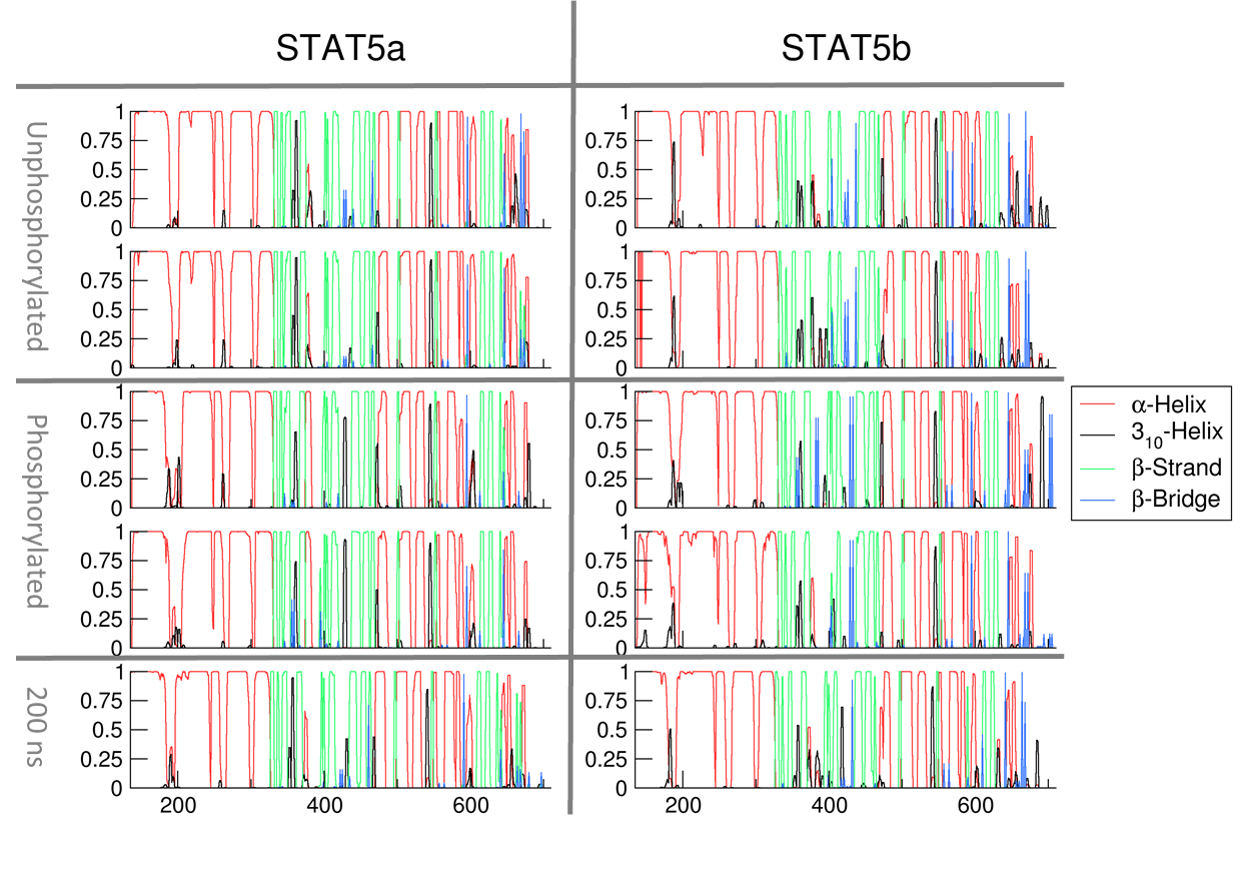

Supplement: S5 Fig — Secondary structure assignments for the STAT5 proteins, focusing on α-helix, 310-helix, β-strand and β-bridge separately. We compare the differences between the STAT5a and STAT5b isoforms (blue and yellow lines, top panels), and between phosphorylated and unphosphorylated STAT5 (dark green and salmon lines, bottom panels). The significant differences (i.e., > standard-deviation) in secondary structure of STAT5 are indicated by red arrows. (TIF) [file pone.0145142.s005.tif]

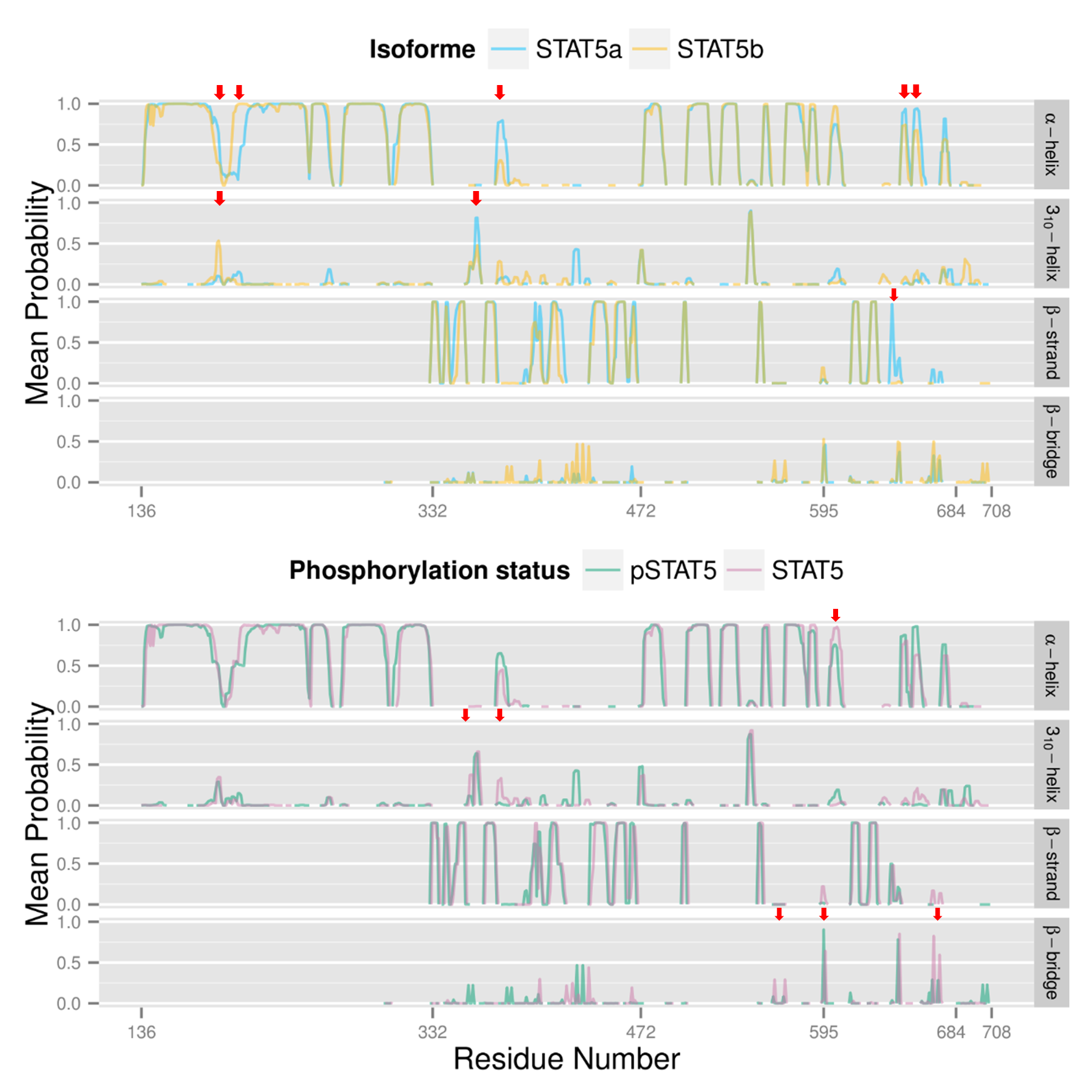

Supplement: S6 Fig — Secondary structures assignment for the STAT5 proteins over the two replicas of MD simulations over the 30- and 200-ns trajectories. For each residue, the proportion of secondary structure type is given as a percentage of the total simulation time and shown with lines of different colour: α-helix is in red, 310-helix is in black, β-sheet is in green, and β-bridge is in blue. (TIF) [file pone.0145142.s006.tif]

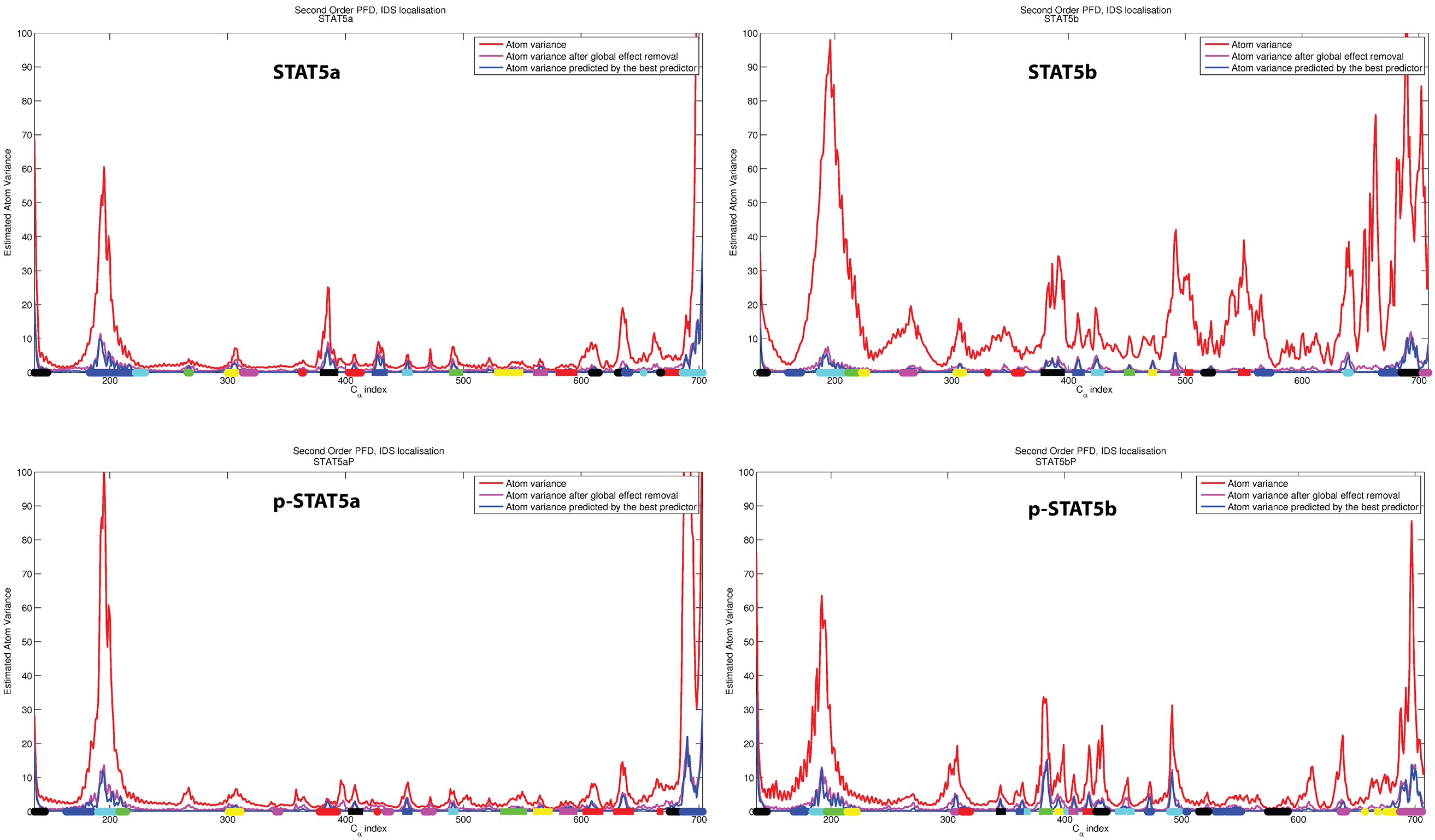

Supplement: S7 Fig — Atomic variance, atomic variance after global removal (q = 6) and atomic variance predicted by the best predictor are shown in red, in violet and in blue respectively. Independent Dynamics Segments (IDSs) are shown as colored sequence segments on the X-axis. (TIF) [file pone.0145142.s007.tif]

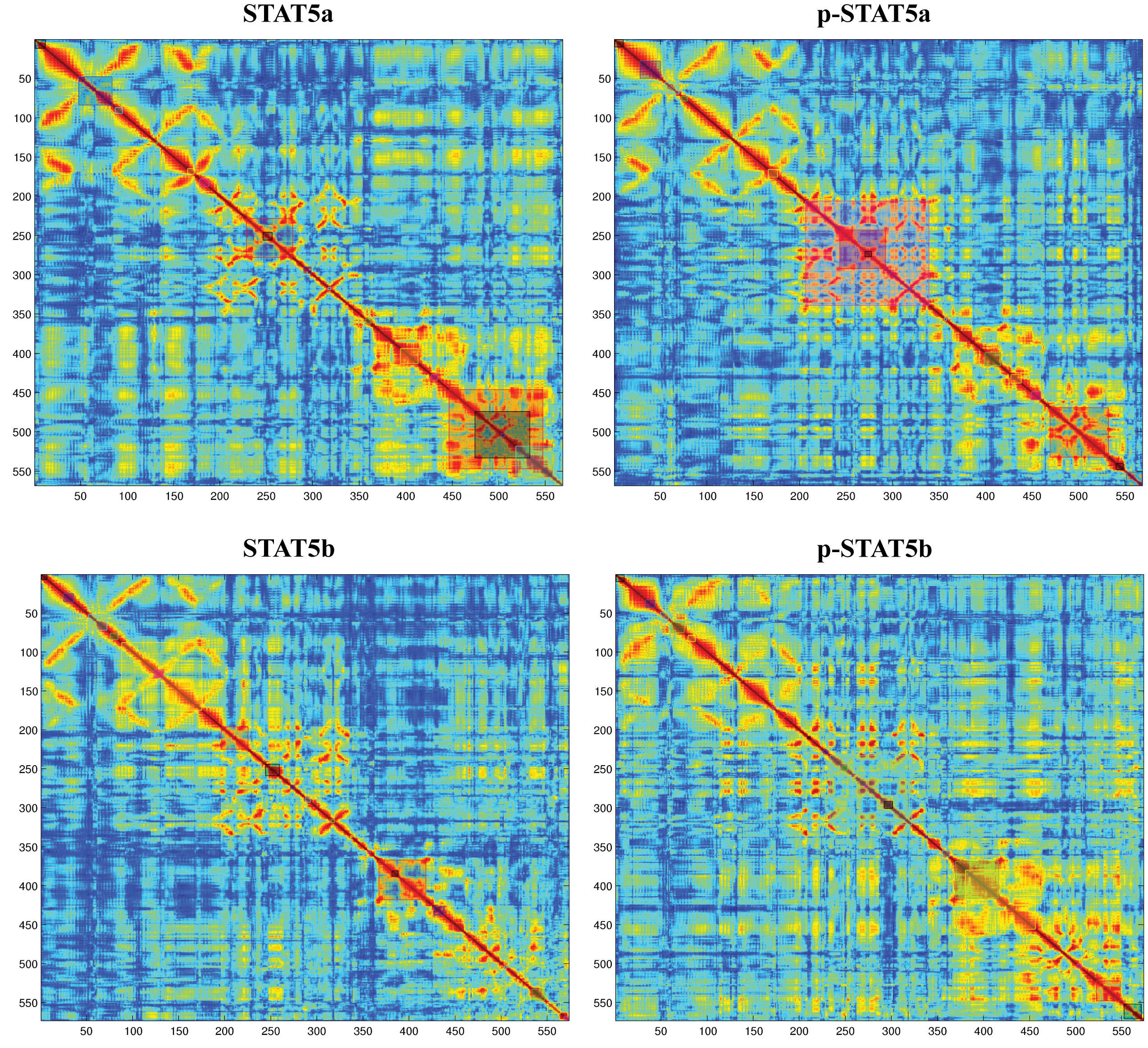

Supplement: S8 Fig — Each IDS in STAT5a, p-STAT5a, STAT5b and STAT5b is presented by a minimal covering square. The heat map represent the residual canonical correlations after removal of the q = 6 slowest PCA modes. (TIF) [file pone.0145142.s008.tif]

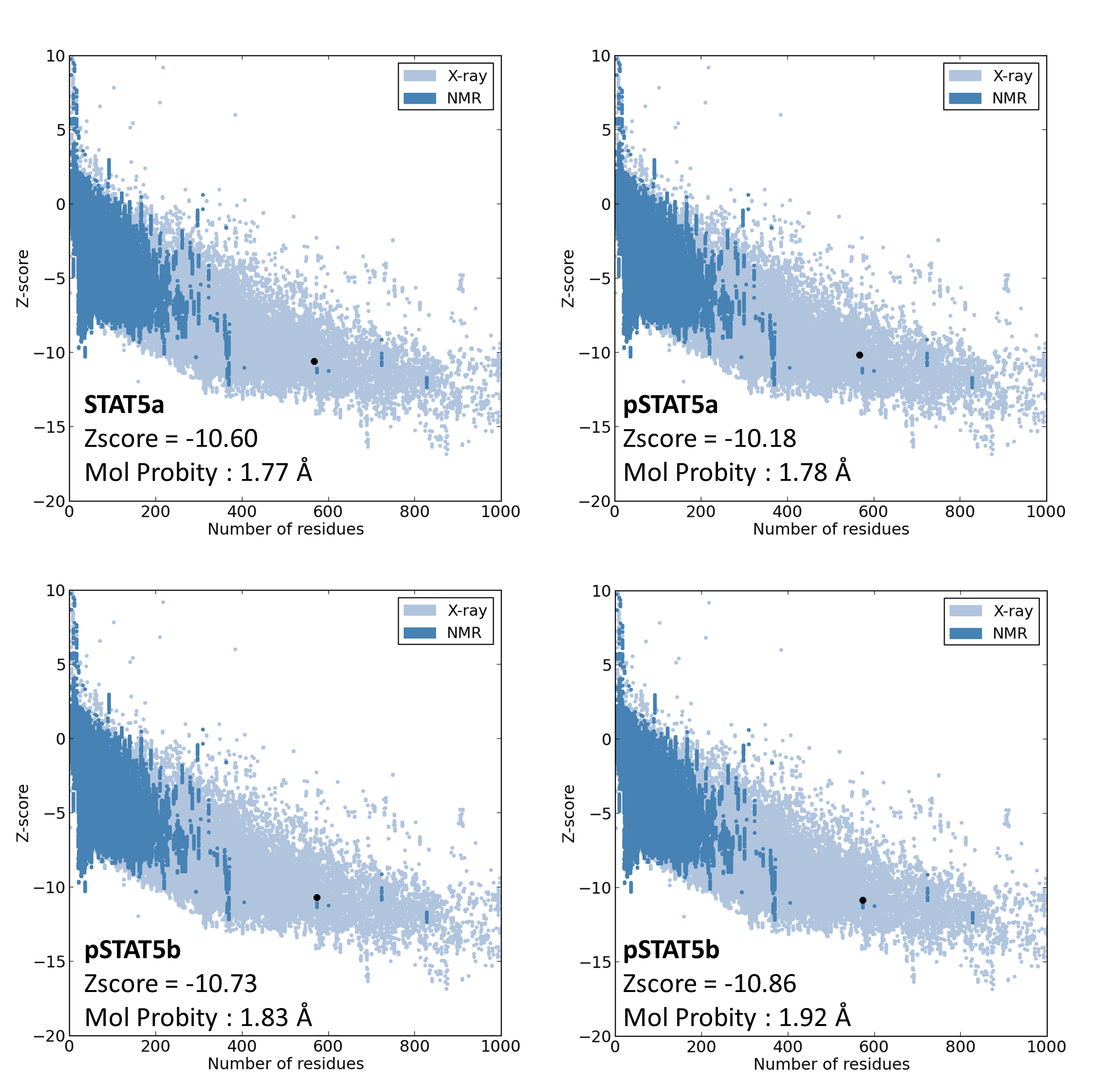

Supplement: S9 Fig — The ProSa-web outputs are shown for STAT5a (upper left), pSTAT5a (upper right), STAT5b (lower left) and pSTAT5b (lower right), assessing the overall model quality. The black points indicate the models compared to the PDB X-Ray (light blue) and NMR (deep blue) structures. The ProSa z-score and the model resolution as determined by MolProbity are shown in the bottom left corner of each panel. (TIF) [file pone.0145142.s009.tif]

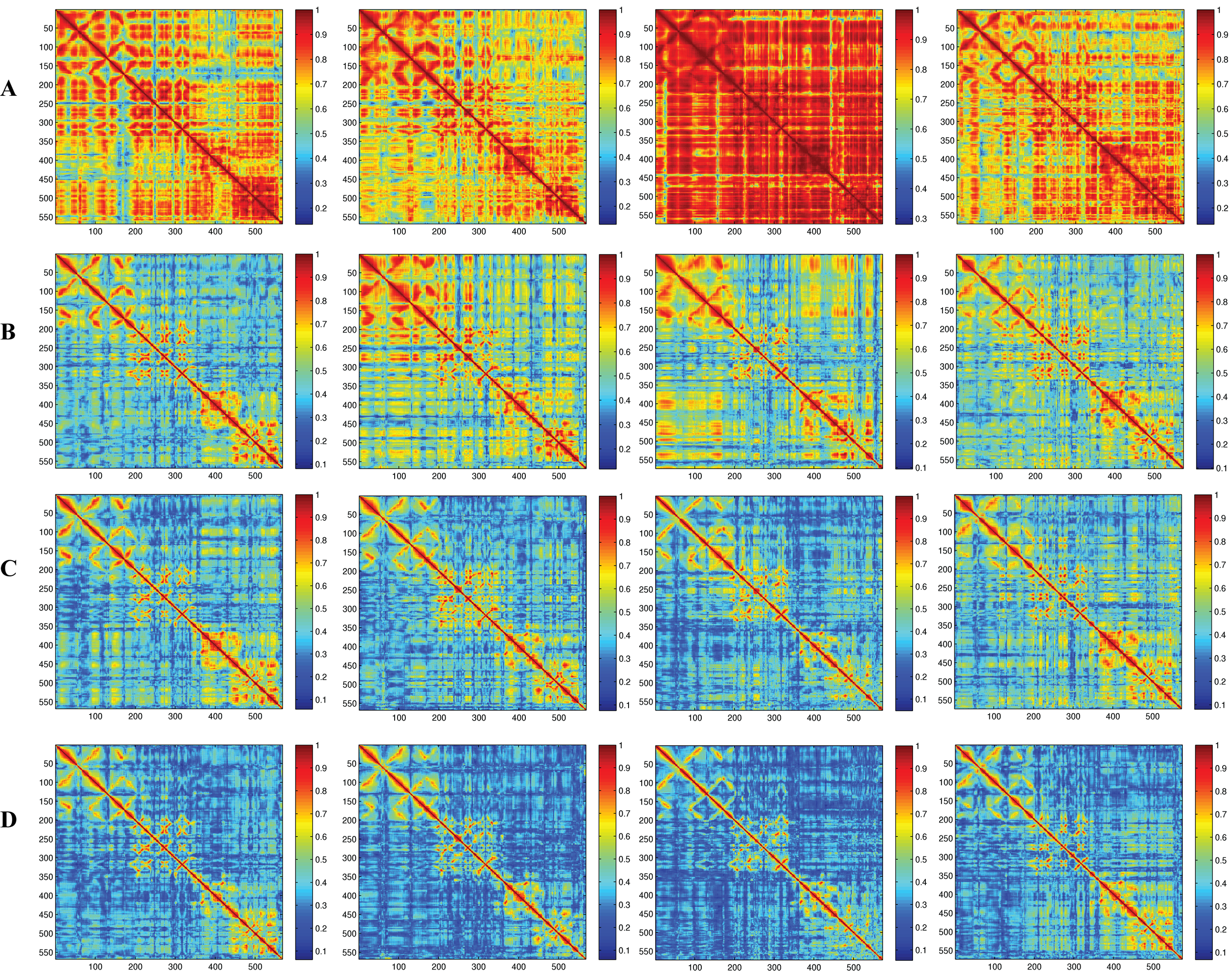

Supplement: S12 Fig — From left to right: STAT5a, p-STAT5a, STAT5b and STAT5b. From (B) to (D): number q of eigenvectors removed (fist row, q = 4; second row, q = 6; third row, q = 8). Correlated motions between Cα atom pairs are presented as color gradient of red (CC = 1) and blue (CC = 0). (TIF) [file pone.0145142.s012.tif]
