# Supplementary material for: How Intrinsic Molecular Dynamics Control Intramolecular Communication in Signal Transducers and Activators of Transcription Factor STAT5
Source: PLoS One. 2015 Dec 30;10(12):e0145142. doi: 10.1371/journal.pone.0145142 (PMC4696835; doi:10.1371/journal.pone.0145142)
Supplement: S1 Table — (PDF) [file pone.0145142.s013.pdf]

S1 Table. The STAT proteins characterized by X-ray or NMR and deposited in the Protein Data Bank (PDB)

PDB Code	STAT protein	Structure composition	Res. (Å)	DNA	Phosphorylation state	Reference**
1BF5 [X-ray]	STAT1 [H]	L136-H182; L197-R683; G700-S710	2.9	Double-strand	Phosphorylated	[12]
1YVL [X-ray]	STAT1 [H]	S2-Q126; T133-E181; A188-N414; I425-K544; K550-Q621; E625-R683	3.0	None	Non-phosphorylated	[13]
2KA6 [NMR]	STAT1 [H]	G706-V750	NA	None	NA	[14]
2KA4 [NMR]	STAT2 [H]	G782-S838	NA	None	NA	[14]
1BG1 [X-ray]	STAT3 [M]	V136-D184; S194-R698 A702-F716	2.3	Double-strand	Phosphorylated	[15]
3CWG [X-ray]	STAT3 [M]	V136-K180; M200-K370 R379-C418; I431-W623; K631-N646; F650-R688	3.1	None	Non-phosphorylated	[16]
4E68 [X-ray]	STAT3 [M]	V136-D184; S194-R688; A702-F716	2.6	Double-strand	Non-phosphorylated	[10]
1BGF [X-ray]	STAT4 [M]	G0*-I123	1.5	None	NA	[17]
1Y1U [X-ray]	STAT5a [M]	S138-R42 ; E433-A690	3.2	None	Non-phosphorylated	[18]
1OJ5 [X-ray]	STAT6 [H]	L795-E808	2.2	None	NA	[19]

H/M indicate Humain/ Mus musculus species.
*Met residue has been replaced by a Gly-Gly numbered 0 and 1.
Structures use for homology modeling are highlight in color.

References**

10. Nkansah E, Shah R, Collie GW, Parkinson GN, Palmer J, Rahman KM, Bui TT, Drake AF, Husby J, Neidle S, Zinzalla G, Thurston DE, Wilderspin AF (2013) Observation of unphosphorylated STAT3 core protein binding to target dsDNA by PEMSA and X-ray crystallography. FEBS Lett 587: 833-839. S0014-5793(13)00110-5 [pii];10.1016/j.febslet.2013.01.065 [doi].
12. Chen X, Vinkemeier U, Zhao Y, Jeruzalmi D, Darnell JE, Jr., Kuriyan J (1998) Crystal structure of a tyrosine phosphorylated STAT-1 dimer bound to DNA. Cell 93: 827-839. S0092-8674(00)81443-9 [pii].

13. Mao X, Ren Z, Parker GN, Sondermann H, Pastorello MA, Wang W, McMurray JS, Demeler B, Darnell JE, Jr., Chen X (2005) Structural bases of unphosphorylated STAT1 association and receptor binding. Mol Cell 17: 761-771. S1097-2765(05)01120-2 [pii];10.1016/j.molcel.2005.02.021 [doi].
14. Wojciak JM, Martinez-Yamout MA, Dyson HJ, Wright PE (2009) Structural basis for recruitment of CBP/p300 coactivators by STAT1 and STAT2 transactivation domains. EMBO J 28: 948-958. emboj200930 [pii];10.1038/emboj.2009.30 [doi].
15. Becker S, Groner B, Muller CW (1998) Three-dimensional structure of the Stat3beta homodimer bound to DNA. Nature 394: 145-151. 10.1038/28101 [doi].
16. Ren Z, Mao X, Mertens C, Krishnaraj R, Qin J, Mandal PK, Romanowski MJ, McMurray JS, Chen X (2008) Crystal structure of unphosphorylated STAT3 core fragment. Biochem Biophys Res Commun 374: 1-5. S0006-291X(08)00691-8 [pii];10.1016/j.bbrc.2008.04.049 [doi].
17. Vinkemeier U, Moarefi I, Darnell JE, Jr., Kuriyan J (1998) Structure of the amino-terminal protein interaction domain of STAT-4. Science 279: 1048-1052.
18. Neculai D, Neculai AM, Verrier S, Straub K, Klumpp K, Pfitzner E, Becker S (2005) Structure of the unphosphorylated STAT5a dimer. J Biol Chem 280: 40782-40787. M507682200 [pii];10.1074/jbc.M507682200 [doi].
19. Razeto A, Ramakrishnan V, Litterst CM, Giller K, Griesinger C, Carlomagno T, Lakomek N, Heimburg T, Lodrini M, Pfitzner E, Becker S (2004) Structure of the NCoA-1/SRC-1 PAS-B domain bound to the LXXLL motif of the STAT6 transactivation domain. J Mol Biol 336: 319-329. S0022283603015687 [pii].

** The reference number is identical to that in manuscript.
