# Supplementary material for: How Intrinsic Molecular Dynamics Control Intramolecular Communication in Signal Transducers and Activators of Transcription Factor STAT5
Source: PLoS One. 2015 Dec 30;10(12):e0145142. doi: 10.1371/journal.pone.0145142 (PMC4696835; doi:10.1371/journal.pone.0145142)
Supplement: S2 Table — The values indicate, for each pair of helices, how many pairs of residues are connected by at least one Communication Pathway, and the total number of Communication Pathways (in brackets). (PDF) [file pone.0145142.s014.pdf]

S2 Table. The inter-residue communication characteristics between helices in CCD. The values indicate, for each pair of helices, how many pairs of residues are connected by at least one *Communication Pathway*, and the total number of *Communication Pathways* (in brackets).

Pair of helices	**STAT5a**	**STAT5b**	**pSTAT5a**	**pSTAT5b**
α1 – α2	71 (1548)	58 (1312)	8 (26)	32 (497)
α1 – α3	114 (5807)	139 (10450)	23 (174)	0 (0)
α1 – α4	0 (0)	0 (0)	1 (1)	0 (0)
α2 – α3	11 (64)	15 (182)	10 (39)	2 (3)
α2 – α4	11 (34)	1 (1)	24 (148)	0 (0)
α3 – α4	12 (26)	0 (0)	0 (0)	0 (0)
Total	219 (7479)	213 (13583)	66 (388)	34 (500)
