# Supplementary material for: How Intrinsic Molecular Dynamics Control Intramolecular Communication in Signal Transducers and Activators of Transcription Factor STAT5
Source: PLoS One. 2015 Dec 30;10(12):e0145142. doi: 10.1371/journal.pone.0145142 (PMC4696835; doi:10.1371/journal.pone.0145142)
Supplement: S3 Table — 1st and 9th 10-quantile values (first and second lines respectively) for the canonical correlation ρ ij before (left) and after (right) removal of the projection on the 6 first eigenvectors (80% of the pairs i ≠ j have a correlation which is between the two values). (PDF) [file pone.0145142.s015.pdf]

S3 Table. **Canonical correlation.** 1st and 9th 10-quantile values (first and second lines respectively) for the canonical correlation $\boldsymbol{\rho_{ij}}$ before (left) and after (right) removal of the projection on the 6 first eigenvectors ($\mathbf{80\%}$ of the pairs $\boldsymbol{i \neq j}$ have a correlation which is between the two values)

Before removal				After removal			
STAT5a	p-STAT5a	STAT5b	p-STAT5b	STAT5a	p-STAT5a	STAT5b	p-STAT5b
0.49	0.50	0.77	0.53	0.27	0.26	0.23	0.29
0.87	0.85	0.97	0.97	0.62	0.61	0.57	0.64
